# Effects of Sulfate Metabolites of Chrysin, Quercetin, Luteolin, and Myricetin on the Albumin Binding of Warfarin (Site I) and Biliverdin (Heme Site): from Theoretical to Practical Considerations

**DOI:** 10.1021/acsomega.6c01121

**Published:** 2026-03-25

**Authors:** Miklós Poór, Lorenzo Pedroni, Dávid Csabai, Dávid Hesszenberger, Anikó Lajtai, Patrik Gömbös, Tamás Huber, Szilvia Barkó, András Lukács, Paul A. Kroon, Kateřina Valentová, Luca Dellafiora, Péter Horváth

**Affiliations:** † Department of Laboratory Medicine, Medical School, University of Pécs, Ifjúság útja 13, Pécs H-7624, Hungary; ‡ Molecular Medicine Research Group, János Szentágothai Research Centre, University of Pécs, Ifjúság útja 20, Pécs H-7624, Hungary; § Department of Food and Drug, 9370 University of Parma, Parco Area delle Scienze 27/A, Parma 43124, Italy; ∥ Agribiotechnology and Precision Breeding for Food Security National Laboratory, Institute of Physiology and Nutrition, Department of Physiology and Animal Health, 37656 Hungarian University of Agriculture and Life Sciences, Guba Sándor u. 40, Kaposvár H-7400, Hungary; ⊥ Department of Biophysics, Medical School, University of Pécs, Szigeti út 12, Pécs H-7624, Hungary; # Food, Microbiome & Health Programme, Quadram Institute Bioscience, Norwich Research Park, Norwich, Norfolk NR4 7UQ, U.K.; ¶ Institute of Microbiology of the Czech Academy of Sciences, Vídeňská 1083, Prague CZ-142 00, Czech Republic; ∇ Department of Pharmaceutical Chemistry, 37637 Semmelweis University, Hőgyes Endre u. 9, Budapest H-1092, Hungary

## Abstract

Serum albumin carries several ligands in the circulation. Previous studies demonstrated that certain flavonoid aglycones and their sulfate metabolites can influence the albumin binding of Site I ligand drugs. To get a deeper insight, the effects of chrysin, quercetin, luteolin, myricetin, and their sulfate derivatives on the albumin binding of warfarin (Site I) and biliverdin (heme site) were examined. Ultracentrifugation experiments were carried out using human serum albumin (HSA), bovine serum albumin (BSA), human serum, and fetal bovine serum (FBS). In addition, fluorescence spectroscopic and modeling studies (warfarin–HSA) as well as circular dichroism-based measurements (biliverdin–HSA) were also performed. Chrysin-7-*O*-sulfate and quercetin-3′-*O*-sulfate considerably displaced warfarin from HSA, while warfarin–BSA interaction was strongly disturbed by luteolin-3′-*O*-sulfate and quercetin-3′-*O*-sulfate. Quercetin-3′-*O*-sulfate, luteolin-3′-*O*-sulfate, and chrysin-7-*O*-sulfate caused the marked displacement of biliverdin from HSA. In contrast, quercetin-3′-*O*-sulfate and luteolin-3′-*O*-sulfate significantly increased the stability of biliverdin–BSA complex. Flavonoid sulfates did not affect the free fraction of warfarin and biliverdin in spiked human serum samples. However, in the presence of 10% FBS (as in cell culture media), high flavonoid levels elevated the free fraction of warfarin, while certain flavonoids considerably decreased the free fraction of biliverdin. Our results demonstrate the complex modulation of warfarin–albumin and biliverdin–albumin interactions by flavonoids. It likely does not influence the albumin binding of Site I and heme site ligands in the human circulation, but flavonoids can strongly affect ligand–BSA interactions in cell culture media.

## Graphical abstract

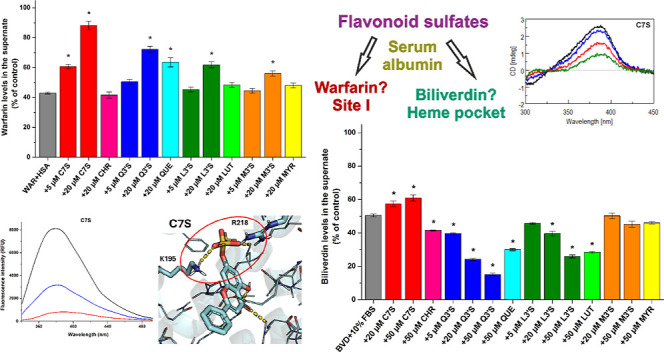

## 1 Introduction

Human serum albumin (HSA) is a multidomain macromolecule containing 585 amino acids (molecular weight ≈66.5 kDa). The protein is built up from three domains (I, II, and III), and each domain includes two subdomains (A and B).^1^ HSA is the most abundant plasma protein in human circulation (35–50 g/L ≈ 525–750 μM) and is responsible for maintaining colloid osmotic (oncotic) pressure in the blood. Furthermore, HSA has buffering, antioxidant, and enzymatic functions and can carry several endogenous molecules and xenobiotics in the circulation.^1^ Generally, the strong interaction of a ligand with HSA results in a “depot effect” due to the high albumin-bound and consequently low unbound free plasma concentration of the molecule, which can make slower the tissue uptake and/or decrease the clearance of the ligand from the body.^1,2^ Importantly, tissue distribution and elimination processes are affected by several other factors and mechanisms; therefore, albumin binding should be considered only one important piece of the pharmacokinetic puzzle. HSA can also be utilized as a drug carrier to optimize drug delivery, including targeted drug therapy.^3,4^ Although a total of nine binding sites of fatty acids on HSA have been identified (FA1–FA9),^1^ drugs and other xenobiotics interact mainly with the following binding sites: Sudlow’s site I (or Site I; FA7; in subdomain IIA), Sudlow’s site II (or Site II; FA3–FA4; in subdomain IIIA), and/or heme pocket (or heme site; FA1; in subdomain IB).^2,5^

Flavonoids are natural polyphenols: aglycones and/or their glycoside derivatives are widely distributed in nature.^6,7^ Typically, flavonoids have low toxicity and can interact with numerous biomolecules in the body; therefore, their potential therapeutic utilization and suitability have been extensively studied for the treatment of inflammatory, cardiovascular, neurodegenerative, and several other diseases.^6^ Most flavonoids have poor oral bioavailability because of their limited solubility in gastrointestinal fluids and high presystemic biotransformation in enterocytes and/or hepatocytes.^8,9^ As a result of the significant first-pass metabolism, most commonly *O*-sulfate and/or *O*-glucuronide conjugates of flavonoids are the dominant forms in circulation,^8^ as demonstrated in healthy human volunteers for chrysin (CHR), quercetin (QUE), and luteolin (LUT).^10–12^ CHR and LUT are flavone aglycones, while QUE and myricetin (MYR) are flavonol aglycones (Figure 1). In addition to the normal dietary intake of these flavonoids (estimated to be around 1–15 mg/day^13^), CHR, QUE, LUT, and MYR are also the major constituents of certain dietary supplements (usually with 100–1000 mg doses in a single tablet/capsule).^14–16^ Earlier studies have demonstrated the strong interactions of these flavonoids with HSA, suggesting that their high-affinity binding site is located in the Site I region.^17–19^ Furthermore, chrysin-7-*O*-sulfate (C7S), quercetin-3′-*O*-sulfate (Q3′S), luteolin-3′-*O*-sulfate (L3′S), and myricetin-3′-*O*-sulfate (M3′S) form more stable complexes with HSA compared to the corresponding parent aglycones, while glucuronide metabolites typically bind to the protein with a considerably lower affinity.^14,16,20,21^ Based on ultrafiltration experiments, these sulfate derivatives can disturb the albumin binding of the Site I marker drug warfarin (WAR), where C7S and Q3′S showed particularly strong displacing effects.^14,16,20,21^ Nevertheless, as a weakness of ultrafiltration, the filtered concentration is not equal to the free concentration of WAR in the sample; therefore, it does not provide direct data. Since Site I and heme site are allosterically coupled,^1^ it is reasonable to hypothesize that flavonoids may affect the albumin binding of heme site ligands as well.

**Figure 1:**
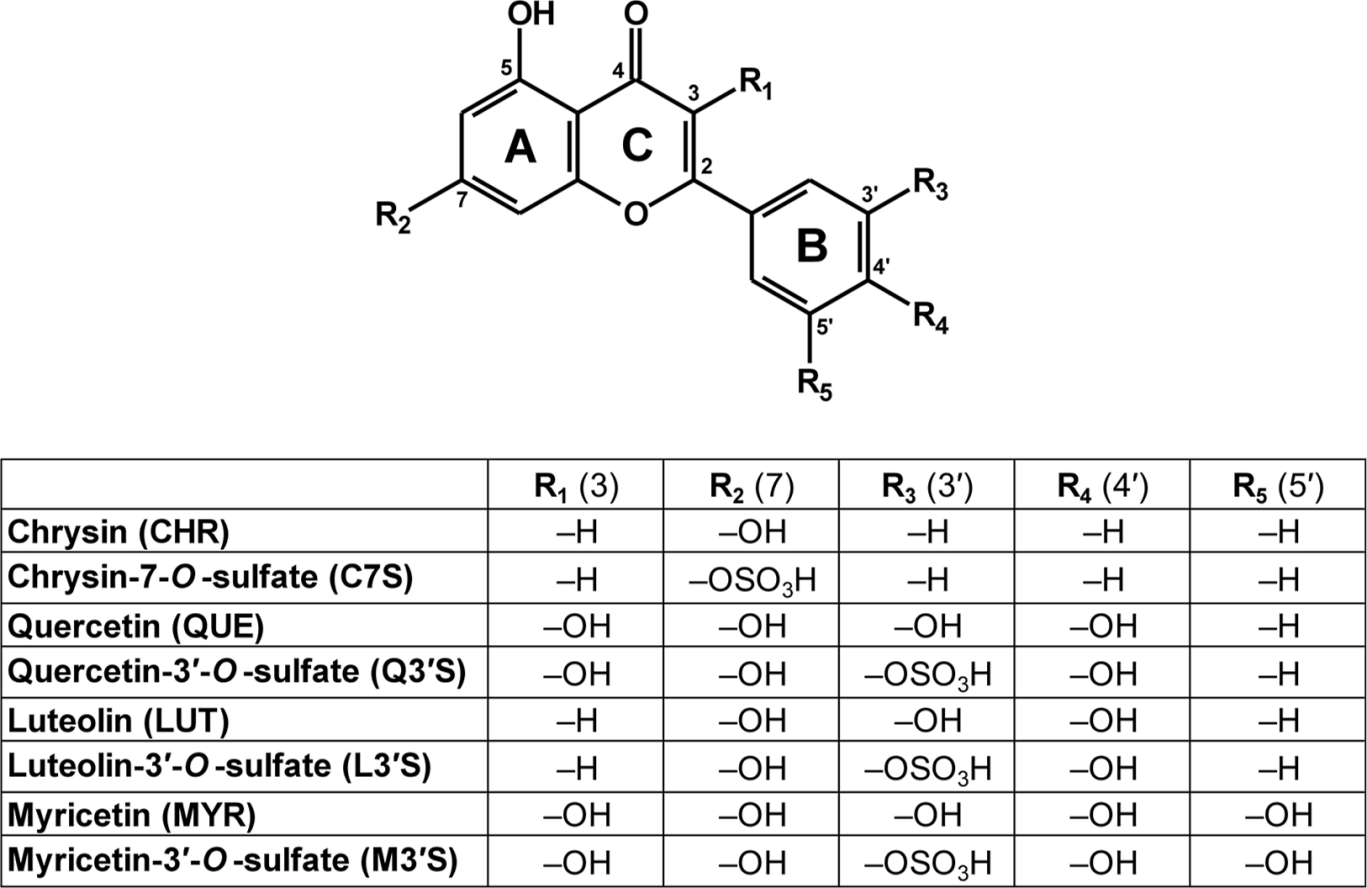
Chemical structures of chrysin, chrysin-7-*O*-sulfate, quercetin, quercetin-3′-*O*-sulfate, luteolin, luteolin-3′-*O*-sulfate, myricetin and myricetin-3′-*O*-sulfate.

In this study, we aimed to investigate the effects of CHR, C7S, QUE, Q3′S, LUT, L3′S, MYR, and M3′S (Figure 1) on the albumin binding of WAR (Site I) and biliverdin (BVD; heme site). Ligand–albumin interactions can be examined using several analytical tools and methodologies, including UV–vis-, fluorescence-, and circular dichroism spectroscopy, surface plasmon resonance, calorimetry, capillary electrophoresis, affinity chromatography, equilibrium dialysis, ultrafiltration, and ultracentrifugation.^22,23^ In the current work, we decided to apply ultracentrifugation as the main technique because, after the gentle sedimentation of ligand–HSA complexes, we can directly measure the free fraction of ligands in the supernate. Even if ultracentrifugation is a low-throughput method and very time-consuming, it is not susceptible to membrane-associated impacts (e.g., Donnan effect and membrane adsorption) which occur during ultrafiltration and equilibrium dialysis,^22,23^ and which can be especially problematic when more than one ligand is added to the protein. First, the impacts of flavonoids were tested in WAR–HSA and BVD–HSA models, then the practical importance of these interactions was also examined with spiked human serum and with 10% fetal bovine serum (FBS; to mimic the effects in cell culture media). Modeling studies were also performed to evaluate flavonoid–HSA interactions. In addition, to compare the results of spectroscopic methods with ultracentrifugation experiments, fluorescence spectroscopy^24^ and circular dichroism^5^ models were applied to test the impacts of flavonoids on WAR–HSA and BVD–HSA interactions, respectively.

## 2 Materials and Methods

### 2.1 Reagents

Chrysin (CHR), quercetin (QUE), luteolin (LUT), racemic warfarin (WAR), biliverdin hydrochloride (BVD), phenylbutazone, furosemide, iodipamide, human serum albumin (HSA; product code: A1653), bovine serum albumin (BSA; product code: A2153), and human serum (product code: H4522) were from Merck (Darmstadt, Germany). Fetal bovine serum (FBS) was obtained from Biosera (Cholet, France; product code: FB-1090). Myricetin (MYR) was purchased from abcr GmbH (Karlsruhe, Germany). Chrysin-7-*O*-sulfate (C7S),^14^ quercetin-3′-*O*-sulfate (Q3′S),^25^ luteolin-3′-*O*-sulfate (L3′S),^26^ and myricetin-3′-*O*-sulfate (M3′S)^27^ were synthesized as it has been previously reported. To mimic extracellular physiological conditions, ultracentrifugation and spectroscopic studies were performed in phosphate-buffered saline (PBS; 137 mM NaCl, 2.7 mM KCl, 10 mM Na_2_HPO_4_, and 1.8 mM KH_2_PO_4_ in water; pH 7.4).

### 2.2 Ultracentrifugation Studies

Ultracentrifugation experiments were carried out as reported earlier.^28–30^ Briefly, samples were centrifuged for 16 h at 170,000*g* and 20 °C, using an Optima MAX-XP ultracentrifuge (Beckman Coulter, Brea, CA, US). Thereafter, WAR or BVD levels were quantified from the protein-free supernates (see details in 2.3). The binding/association constants (*K*) of ligand–protein complexes were calculated assuming 1:1 stoichiometry^28,30,31^

1 $$K=\frac{\left[L-SA\right]}{\left[L\right]\times \left[SA\right]}$$

where *[L]* is the molar concentration of the unbound free ligand, *[*SA*]* denotes the molar concentration of the unbound free albumin, and *[L–*SA*]* means the molar concentration of the ligand–albumin complex. In ultracentrifugation experiments, the recovery of the ligands examined were the following (mean ± SEM values): 98.2 ± 1.7% for WAR and 94.6 ± 1.5% for BVD.

### 2.3 Chromatographic Analyses

After ultracentrifugation, WAR was quantified in the protein-free supernates using a Shimadzu UHPLC–FLD system (Kyoto, Japan), including an autosampler (Nexera X2 SIL-30AC), a degasser (DGU-20A5R), the pump modules (Nexera X2 LC30AD), a column oven (CTO-20AC), a fluorescence detector (RF-20A XS), and the LabSolutions 5.97 SP1 software. The isocratic elution was carried out employing sodium phosphate buffer (20 mM, pH 7.0), methanol, and acetonitrile (70:25:5 v/v %) as the mobile phase, with 1.0 mL/min flow rate (injected volume: 20 μL; column temperature: 24 °C). Samples were driven through a precolumn (SecurityGuard EVO-C18 ULTRA; Phenomenex, Torrance, CA, USA) linked to a Kinetex EVO C18 analytical column (150 × 4.6 mm, 2.6 μm particle size, pore size 100 Å; Phenomenex). WAR was detected at 390 nm (λ_ex_ = 310 nm). The major validation parameters of the method were the following: linearity (0.01–5.0 μM), *R* ^*2*^ = 0.999; limit of detection (LOD, signal-to-noise ratio of 3), 0.01 μM; limit of quantification (LOQ, signal-to-noise ratio of 10), 0.03 μM; intraday precision (*n* = 7), 0.8%.

After ultracentrifugation, BVD was quantified in the protein-free supernates using an integrated Jasco HPLC–UV system (Tokyo, Japan), including an autosampler (AS-4050), a binary pump (PU-4180), an UV detector (UV-975), and the ChromNAV2 software. Without further sample preparation, the supernates (20 μL) were driven through a precolumn (Security Guard C18, 4.0 × 3.0 mm; Phenomenex) linked to a Gemini C18 (100 × 4.6 mm, 5 μm; Phenomenex) analytical column. The isocratic elution was performed with sodium acetate buffer (20 mM, pH 5.0) and methanol (25:75 v/v %) with 1 mL/min flow rate at room temperature. BVD was detected at 380 nm. The major validation parameters of the method were the following: linearity (0.05–5.0 μM), *R* ^*2*^ = 0.999; LOD, 0.03 μM; LOQ, 0.10 μM; intraday precision (*n* = 7), 0.9%.

LC–MS analyses of the ultracentrifuged BVD serum samples were performed with a Prominence HPLC system coupled with a Shimadzu LCMS-2020 single quadrupole mass spectrometer (Kyoto, Japan). Samples (10 μL) were driven through a Kinetex XB-C18 analytical column (100 mm × 2.1 mm, 2.6 μm particle size; Phenomenex) guarded by a KrudKatcher ULTRA precolumn (0.1 mm, 2 μm particle size; Phenomenex) with 0.2 mL/min flow rate. Column thermostat was set to 30 °C. Gradient elution was applied using water with 0.1 v/v % formic acid and 5 mM ammonium formate as eluent A and methanol as eluent B. Eluent B was increased linearly from 10% to 60% in 3 min, then to 100% in an additional 8 min. Thereafter, the column was washed with 100% eluent B for 3 min, set back to the initial 10% within 1 min, and re-equilibrated for 3 min. Ionization of BVD was performed using an ESI ion source in positive SIM mode. The three most intense *m*/*z* (583.25, 584.20, and 621.20) were used, the former as a quantitative and the least two as confirmatory ions. Major validation parameters: linearity (0–200 nM), *R* ^*2*^ = 0.999; LOD, 0.002 μM; LOQ, 0.006 μM; intraday repeatability (*n* = 7), 3.0%.

### 2.4 Modeling Studies

The 3D structure of HSA cocrystallized with WAR was obtained from the Protein Data Bank (PDB) (https://www.rcsb.org/)^32^ under the PDB ID 2BXD.^33^ The 3D structure was processed removing all cocrystallized molecules—WAR, water, and ions—while only keeping the protein structure. The 3D structures for the flavonoids were obtained from PubChem (https://pubchem.ncbi.nlm.nih.gov/)^34^ in the SDF format under the following CIDs: CHR (5281607), C7S (69503899), QUE (5280343), Q3′S (5280362), LUT (5280445), L3′S (44258151), MYR (5281672), and WAR (54678486). M3′S was not available on PubChem, it was thus made using the PyMol Builder tool (v. 2.5.0) properly editing the MYR 3D structure. SDF to Mol2 conversion, and correct charges assignment (pH = 7) were performed using Obabel^35^ with WAR and all the flavonoids tested.

Molecular docking simulation was performed through GOLD (Genetic Optimization for Ligand Docking; v. 2024) to provide the most plausible architecture of binding for the set of considered flavonoids (the higher the docking score, the most plausible the binding architecture, as per manufacturer declaration; https://www.ccdc.cam.ac.uk). The binding site was defined within a 10 Å radius sphere around the centroid of the WAR binding site (as per PDB ID 2BXD). Each ligand was kept fully flexible while for HSA only polar hydrogens were set to rotate freely, as per previous study.^36^ The crystallographic pose of WAR derived from PDB ID 2BXD was used to set hydrogen-bond donor/acceptor restraints to better inform the docking procedure. The internal PLP scoring function was adopted to rank the obtained poses for each ligand tested.

### 2.5 Fluorescence Spectroscopic Studies

Fluorescence emission spectra were recorded using a Hitachi F-4500 fluorometer (Tokyo, Japan) applying 317 nm excitation wavelength, then the flavonoid-induced changes in the emission signals were evaluated at 380 nm (excitation and emission wavelength maxima of HSA-bound WAR).^24^ Measurements were carried out in standard 1 cm × 1 cm cuvettes at 25 °C, applying 90-degree fluorescence detection. The inner-filter effects of flavonoids were corrected based on the following equation^37^

2 $$F_{cor}=F_{obs}\times 10^{\left(A_{ex}+A_{em}\right)/2}$$

where *F*_cor_ and *F*_obs_ are the corrected and the observed fluorescence at 380 nm, respectively; while *A*_ex_ and *A*_em_ are the absorbance of flavonoids at 317 and 380 nm, respectively. Since albumin-bound WAR exerts more than 15-fold stronger emission signal than the free fluorophore,^24^ the flavonoid-induced decrease in the bound fraction of WAR may correlate with the displacing effect.

### 2.6 Circular Dichroism Studies

CD and UV spectra were measured in parallel on a J-815 spectropolarimeter (Jasco, Tokyo, Japan) at 25 ± 0.05 °C in a 1 cm optical path length rectangular quartz cell (Hellma, Müllheim, Germany). The temperature was controlled using a Jasco CDF-426L Peltier thermostat. All spectra were registered using the following parameters: range = 250–450 nm; data pitch = 0.1 nm; standard sensitivity; data integration time (D.I.T.) = 1 s; bandwidth = 2.0 nm; scanning speed = 50 nm/min; and accumulations = 3. To correct for cuvette characteristics, the spectrum of the cuvette containing the solvent (PBS) was initially recorded and then subtracted it from the spectra of the samples. Spectra were processed using Jasco Spectra Analysis software (Spectra Manager Version 2.15.09 [Build 1]).

### 2.7 Statistical Analyses

Data represent means ± standard errors of the mean (SEM) values at least from three independent experiments. Statistical evaluation was performed by one-way ANOVA and Tukey’s posthoc tests using SPSS Statistics software (version 24.0; IBM, Armonk, NY, US), where the level of significance was set to *p* < 0.01.

## 3 Results

### 3.1 Effects of Flavonoids on WAR–HSA Interaction Based on Ultracentrifugation, Fluorescence Spectroscopic, and Modeling Studies

Flavonoid sulfates (5 or 20 μM) and flavonoid aglycones (20 μM) were added to standard concentrations of WAR (1 μM) and HSA (5 μM). After ultracentrifugation, WAR was directly quantified from the protein-free supernates with UHPLC–FLD. In the absence of flavonoids, WAR levels were approximately 43% in the supernate (corresponds to the free fraction of the Site I marker). Among the flavonoid aglycones, only QUE caused a statistically significant (*p* < 0.01) elevation in the free concentration of WAR, while LUT, MYR, and CHR showed only minor or no impacts (Figure 2A). In contrast, each sulfate metabolite markedly increased WAR levels, demonstrating stronger effects compared to their parent aglycones. The largest changes were induced by C7S (which also had considerable effect even at 5 μM concentration), then it was followed by Q3′S, L3′S, and M3′S (Figure 2A).

**Figure 2:**
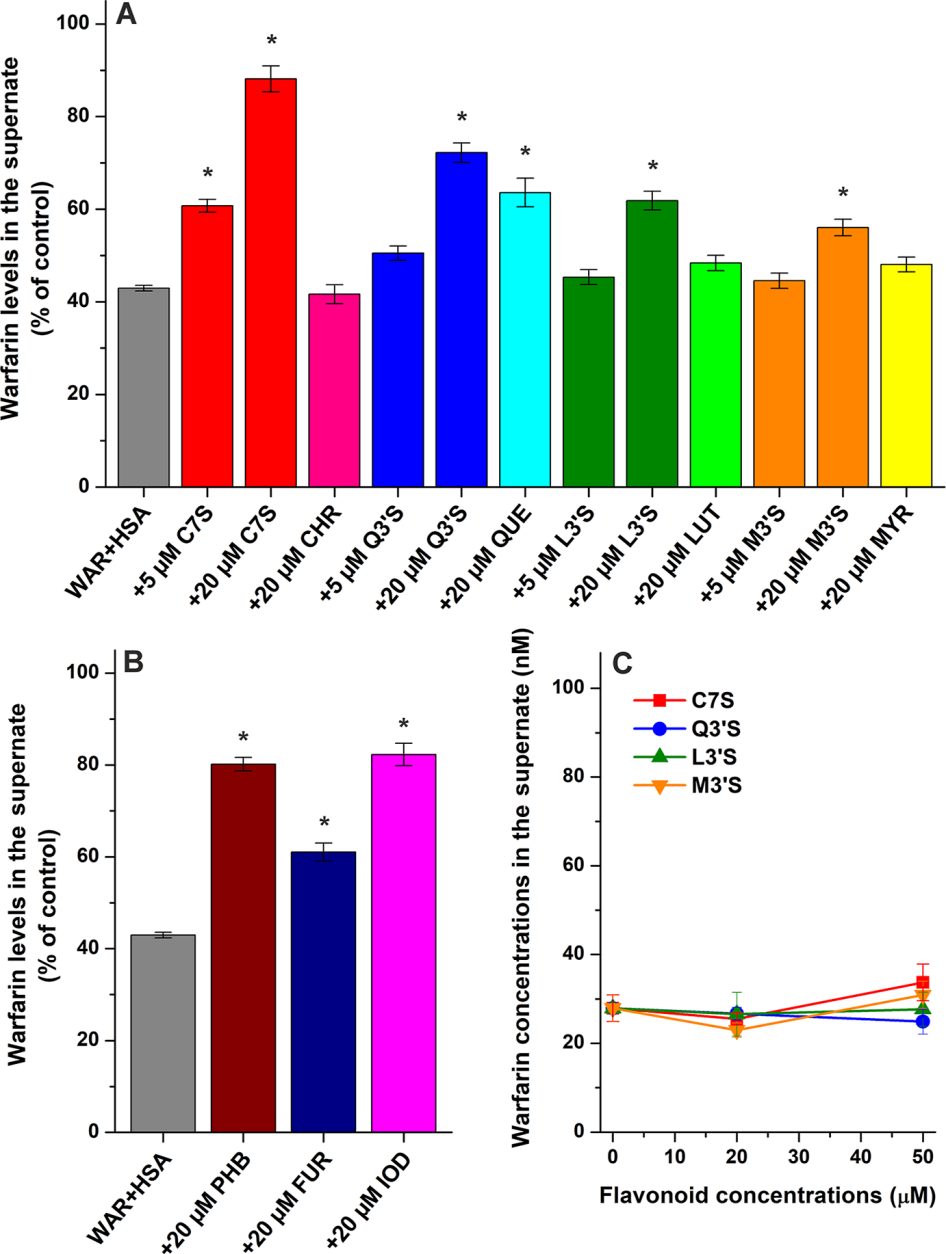
WAR (1 μM) levels in the supernates after ultracentrifugation (16 h, 170,000*g*, 20 °C) in the presence of HSA (5 μM) without and with C7S, CHR, Q3′S, QUE, L3′S, LUT, M3′S, or MYR in PBS (pH 7.4) (A). WAR (1 μM) levels in the supernates after ultracentrifugation in the presence of HSA (5 μM) without and with Site I marker drugs phenylbutazone (PHB), furosemide (FUR), or iodipamide (IOD) in PBS (B). Panels **A** and **B** demonstrate changes in WAR levels compared to WAR (1 μM) ultracentrifuged without the protein and other ligands (100%). WAR (5 μM) concentrations in the supernates after ultracentrifugation of spiked human serum samples without and with C7S, Q3′S, L3′S, or M3′S (C). Data represent means ± SEM from three independent experiments (*p < 0.01).

To do a comparison with well-known high-affinity Site I ligands, the impacts of phenylbutazone, furosemide, and iodipamide (each 20 μM) were also tested. Under the applied conditions, these ligands caused large increases in free WAR levels (Figure 2B). Nevertheless, even compared to iodipamide and phenylbutazone, C7S demonstrated stronger impact. Q3′S induced somewhat lower changes than these two drugs, while its displacing effect was higher compared to furosemide. QUE, L3′S, and M3′S showed similar impacts to furosemide.

Considering the stronger displacing ability of sulfate conjugates vs their aglycones and the typically very low levels of flavonoid aglycones in the circulation, the effects of C7S, Q3′S, L3′S, and M3′S on the albumin binding of WAR were also examined in spiked human serum samples, where high levels of flavonoids (final concentrations: 20 or 50 μM) were added to a realistic (therapeutic) concentration of WAR (5 μM).^38^ After ultracentrifugation, we found the free serum level of WAR around 28 nM, where even the highest concentrations of flavonoid metabolites examined did not affect significantly (*p* < 0.01) the albumin binding of the Site I marker drug (Figure 2C).

Based on the albumin-induced changes in the free fractions of WAR quantified in the ultracentrifugation experiments, the binding constant (*K*; unit: L/mol) of WAR–HSA complex was calculated using eq 1. We determined 5.46 ± 0.02 as the log*K* value of WAR–HSA, which showed a good correlation with previously reported data.^24,39,40^ Furthermore, approximately 0.6% was measured as the free fraction of WAR in spiked human serum, which is in line with the clinical data (typically between 0.3 and 1.0%).^38^

To make a comparison with the results of ultracentrifugation experiments, we selected a fluorescence spectroscopic model, which applies the principle that the interaction of WAR with HSA leads to a remarkable increase in the emission signal of the Site I marker drug (Figure S1). Thus, the displacement of WAR from HSA should decrease its emission signal. In these experiments, the same concentrations of WAR (1 μM), HSA (5 μM), and flavonoids (5 and 20 μM) were used than in the ultracentrifugation studies (Figure 2A). Representative fluorescence emission spectra are demonstrated in Figure S2 (λ_ex_ = 317 nm). Flavonoids alone and in the presence of HSA did not show relevant background signals at 380 nm (fluorescence emission wavelength maximum of WAR–HSA complex). After correction of the inner-filter effects of flavonoids (based on eq 2), the emission intensities were represented in Figure 3. Each flavonoid induced large decrease in the fluorescence signal of WAR, where C7S caused the largest and CHR/MYR resulted in the smallest changes.

**Figure 3:**
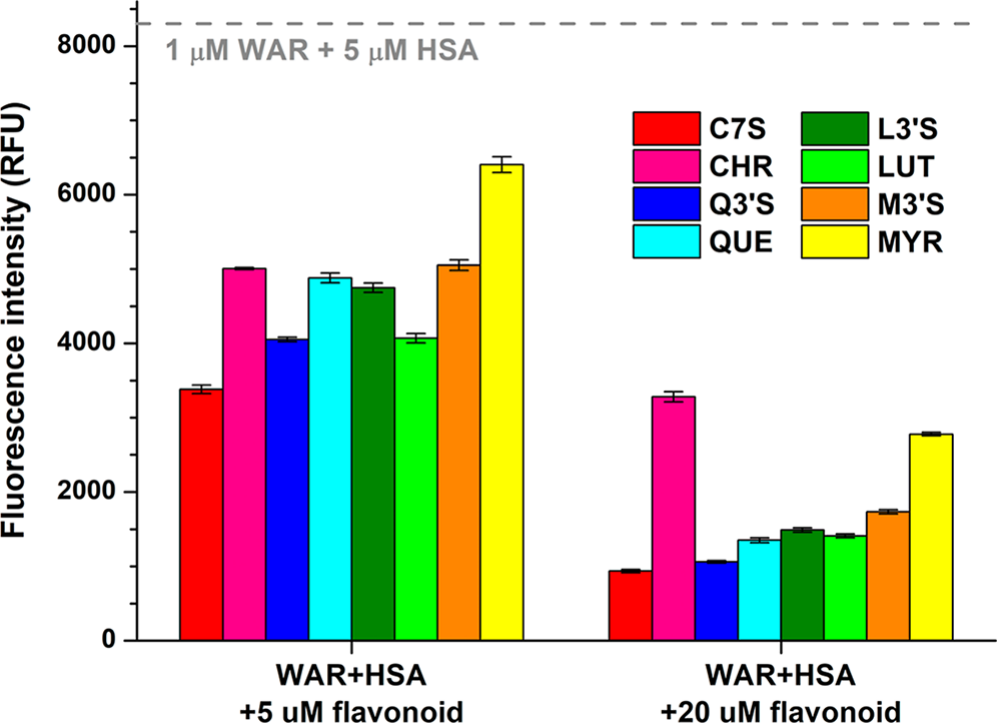
Fluorescence emission intensity of WAR (1 μM) + HSA (5 μM) in the presence of flavonoids (5 μM or 20 μM) in PBS (pH 7.4; λ_ex_ = 317 nm, λ_em_ = 380 nm; the inner-filter effects of flavonoids were corrected based on eq 2; see representative emission spectra in Figure S2).

The binding architecture of WAR and flavonoids (CHR, C7S, QUE, Q3′S, LUT, L3′S, MYR, and M3′S) was investigated through molecular docking simulations performed using GOLD. The docking protocol was first validated and finely tuned using WAR as a reference ligand. Specifically, the cocrystallized pose of WAR from PDB entry 2BXD was employed to optimize the docking parameters and ensure the accurate reproduction of the available crystallographic binding mode (PDB ID 2BXD), as demonstrated in Figure S3. This optimized docking protocol was applied to dock the set of flavonoids and their sulfate derivatives. Each ligand achieved positive and comparable docking scores (Table S1), indicating that the whole set can satisfy the physicochemical properties of the WAR binding pocket. After an expert visual inspection of the calculated binding poses, we observed some interesting differences among flavonoids: C7S, L3′S, and M3′S were able to form additional salt bridges mediated by the sulfate group (interactions not observed in their respective parent compounds), which are known to provide a valuable contribution to the binding energy (Figure 4). In contrast, Q3′S adopted a different orientation that did not display such interactions and maintaining a binding pattern comparable to that of its parent compound. However, under experimental conditions, a salt bridge formation between the sulfate group of Q3′S and Arg218 guanidinium group could occur, given the short interatomic distance observed (<4 Å). Docking studies suggest that the binding site of each flavonoid—at least partially—overlap with the binding pose of WAR (Figure S4).

**Figure 4:**
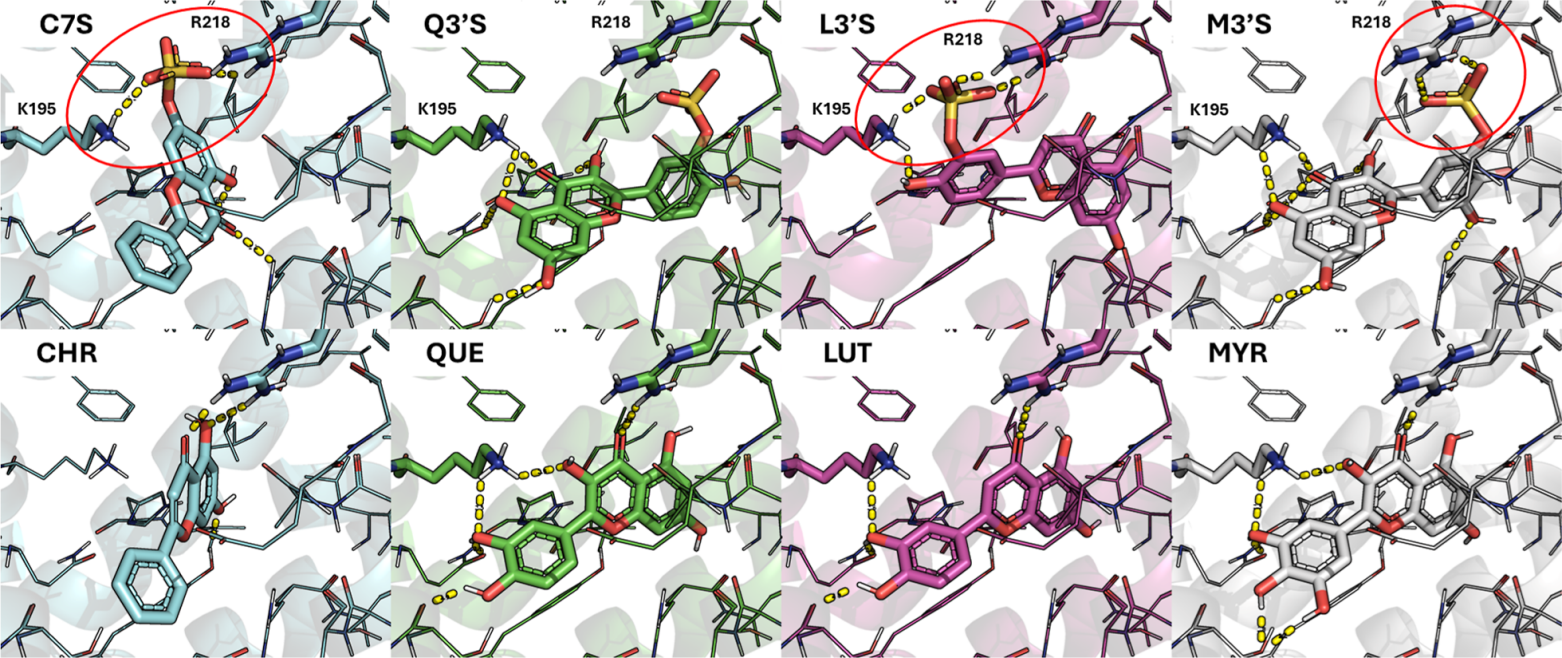
Binding modes of flavonoids and their sulfate derivatives (shown as sticks) within the HSA (shown as transparent white cartoon) binding site after the docking simulation. The panels on top show the interactions of sulfated flavonoids (C7S, Q3′S, L3′S, and M3′S), while their corresponding nonsulfated forms (aglycones: CHR, QUE, LUT, and MYR) are reported in the bottom panels. Key residues involved in salt bridges are shown as sticks, i.e. Lys195 (K195) and Arg218 (R218), and the corresponding salt bridges are embedded by red circles. Other polar contacts are reported as yellow dashed lines. The presence of the sulfate group enhances interactions with R218, suggesting a potential role in modulating binding affinity and specificity.

### 3.2 Effects of Flavonoids on the Albumin Binding of WAR in the Presence of 10% FBS

Our results demonstrated that flavonoids cannot interfere with the albumin-binding of WAR in human serum (Figure 2C). However, in cell experiments, lower amount (10%) of FBS is typically contained by cell culture media, where the presence of flavonoids may affect the interaction of the Site I marker drug with BSA. To test this hypothesis, FBS was diluted to 10-fold with PBS, and the impacts of flavonoids (20 and/or 50 μM) were examined on the free fraction of WAR (5 μM). After ultracentrifugation, FBS (10%) induced approximately 86% decrease in WAR levels of the supernate. Each flavonoid (50 μM) increased the free concentration of WAR (Figure 5): the largest elevation was caused by L3′S, followed by Q3′S. Displacing effects of C7S vs CHR and M3′S vs MYR were similar, while the impacts of L3′S and Q3′S proved to be stronger compared to their parent aglycones (Figure 5).

**Figure 5:**
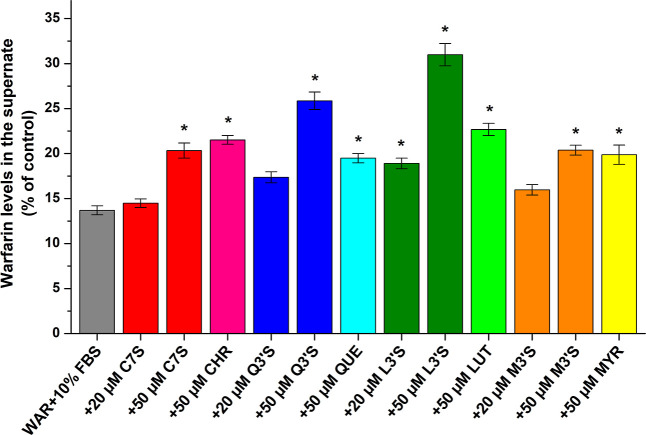
WAR (5 μM) levels in the supernates after ultracentrifugation (16 h, 170,000*g*, 20 °C) in the presence of 10% fetal bovine serum (FBS) without and with C7S, CHR, Q3′S, QUE, L3′S, LUT, M3′S, or MYR in PBS (pH 7.4). Data represent means ± SEM from three independent experiments (*p < 0.01), where the changes in WAR levels were compared to WAR (5 μM) ultracentrifuged without FBS and other ligands (100%).

Since considerable differences were noticed in the displacing effects of flavonoids in the presence of HSA (5 μM; Figure 2A) or FBS (10%; Figure 5), ultracentrifugation experiments were also performed with BSA and flavonoid sulfates, where we noticed again the strongest displacing effects of L3′S and Q3′S (Figure S5). Furthermore, slightly lower stability of WAR–BSA (log*K* = 5.29 ± 0.09) was determined compared to the WAR–HSA complex, which is in accordance with the previously described results based on fluorescence spectroscopic experiments.^41^

### 3.3 Effects of Flavonoids on BVD–HSA Interaction Based on Ultracentrifugation and Circular Dichroism Studies

In the following experiment, flavonoid sulfates (1.5, 5, or 20 μM) and flavonoid aglycones (20 μM) were added to standard concentrations of BVD (1 μM) and HSA (2 μM). After ultracentrifugation, BVD was directly quantified from the protein-free supernates with HPLC–UV. In the absence of flavonoids, BVD levels were approximately 51% in the supernate (corresponds to the free fraction of the heme site marker). At 20 μM concentrations, both flavonoid aglycones and sulfates induced statistically significant (*p* < 0.01) elevation in BVD concentrations of the supernates (Figure 6A). Among the flavonoid aglycones tested, the strongest impact was caused by CHR, followed by LUT, MYR, and QUE. Furthermore, the sulfate metabolites induced larger increases in BVD levels compared to their corresponding parent aglycones (Figure 6A). At 20 μM concentrations, Q3′S and L3′S almost completely abolished the interaction of BVD with HSA. In addition, Q3′S caused significant elevation of free BVD levels even at 1.5 μM concentration (Figure 6A).

**Figure 6:**
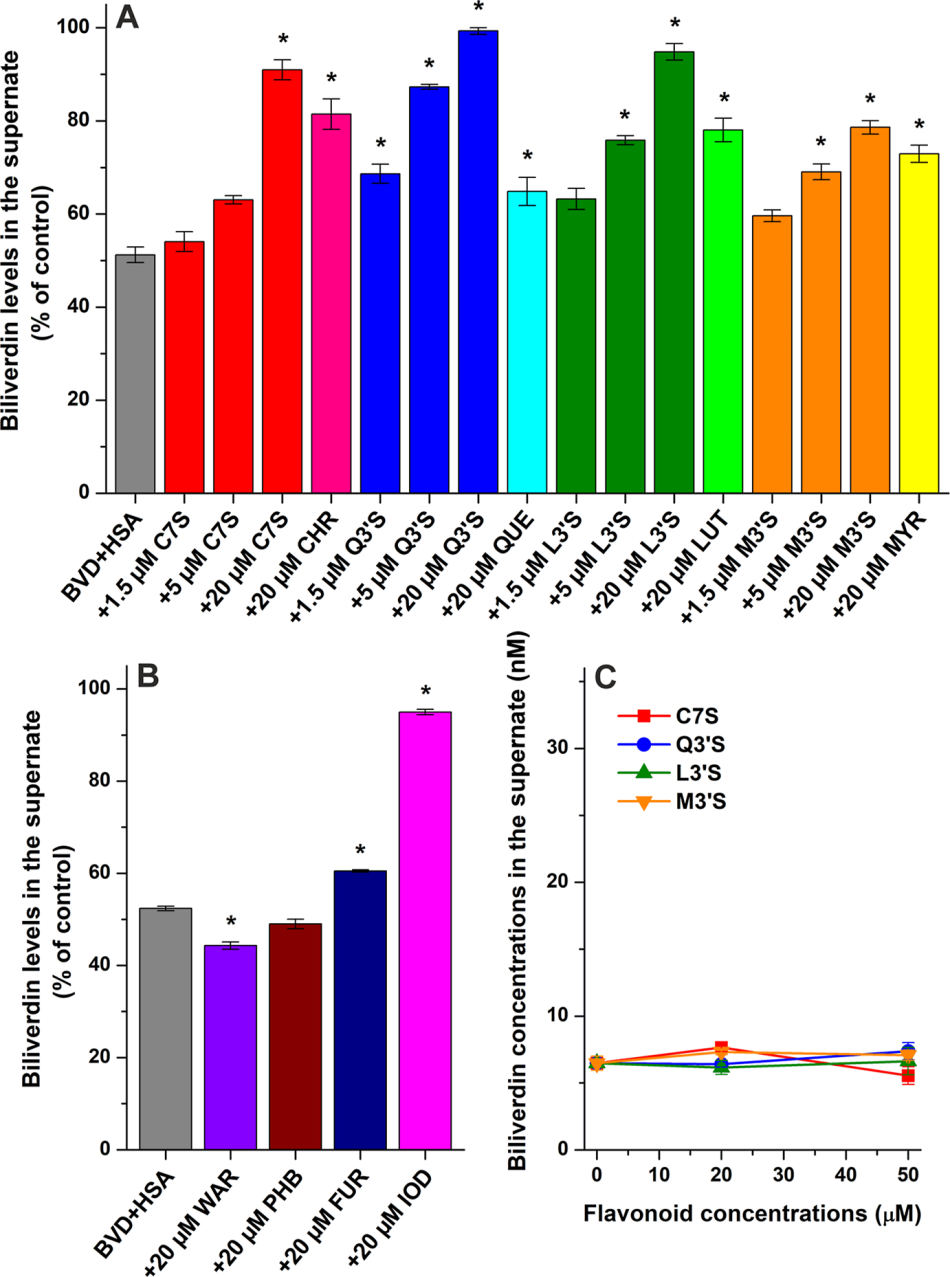
BVD (1 μM) levels in the supernates after ultracentrifugation (16 h, 170,000*g*, 20 °C) in the presence of HSA (2 μM) without and with C7S, CHR, Q3′S, QUE, L3′S, LUT, M3′S, or MYR in PBS (pH 7.4) (A). BVD (1 μM) levels in the supernates after ultracentrifugation in the presence of HSA (2 μM) without and with Site I marker drugs WAR, phenylbutazone (PHB), furosemide (FUR), or iodipamide (IOD) in PBS (B). Panels **A** and **B** demonstrate changes in BVD levels compared to BVD (1 μM) ultracentrifuged without the protein and other ligands (100%). BVD (5 μM) concentrations in the supernates after ultracentrifugation of spiked human serum samples without and with C7S, Q3′S, L3′S, or M3′S (C). Data represent means ± SEM from three independent experiments (*p < 0.01).

The effects of Site I marker drugs (each 20 μM) on BVD–HSA interaction were also examined. Under the applied conditions, phenylbutazone barely affected and WAR significantly (*p* < 0.01) decreased the concentrations of BVD in the supernates (Figure 6B). In contrast, moderate and considerable elevations in free BVD levels were noticed in the presence of furosemide and iodipamide, respectively (Figure 6B). At 20 μM concentrations, Q3′S, L3′S, and C7S caused impacts similar to iodipamide (Figure 6).

The potential effects of flavonoid sulfates on the albumin-binding of BVD were also tested in spiked human serum samples. After ultracentrifugation, the free concentrations of BVD were quantified with LC–MS, which was required due to the very low BVD levels in the supernates. Human serum was spiked with high levels of flavonoids (20 or 50 μM) and with 5 μM concentration of BVD. Using these conditions, we found the free serum level of BVD around 7 nM (≈0.1%), where even the highest concentration of flavonoids examined did not affect the albumin binding of BVD (Figure 6C).

For the spectroscopic investigation of BVD–HSA interaction, a circular dichroism-based model was selected. BVD binds to HSA in subdomain IB (heme site), resulting in a positive Induced Circular Dichroism (ICD) signal within the BVD absorption band range (λ_max_ = 386 nm; see representative CD and UV spectra in Figure S6).^5^ Thus, the displacement of BVD from the protein should decrease its ICD band. We applied the same concentrations of BVD (1 μM), HSA (2 μM), and flavonoids (0–20 μM) than in the ultracentrifugation studies (Figure 6A). Under these conditions, flavonoids reduced the ICD band of BVD in a concentration-dependent fashion, except MYR which caused a minor decrease only at 20 μM concentration (Figure 7). Comparing CHR and C7S, the decline was more pronounced for CHR, and a weak ICD couplet appeared around 280 nm (Figure 7A,B). Conversely, we did not observe an ICD signal in the absorption band around 350 nm, which is characteristic for both flavonoids (Figure S7). This is a significant difference compared to QUE (Figure 7C), for example. Therefore, we assumed that the benzopyran-4-one structure did not interact with the peptide chain and remained unperturbed. The appearance of the ICD couplet at 280 nm is most likely due to perturbation of the carbonyl *n*–π* transition. Regarding QUE and Q3′S, the ICD spectra differ in both the BVD-containing and the BVD-free spectra (Figure S8), and the ICD band of BVD shows decreases and band splitting (Figure 7C,D). The reduction in the intensity was more pronounced for the metabolite, whereas the band splitting was stronger for QUE. Since band splitting also occurs in spectra without BVD, it may result from the interaction of QUE and Q3′S with one of the aromatic chromophore groups of HSA. Furthermore, QUE and Q3′S showed ICD signals in the 275–280 nm range in both BVD-containing and BVD-free HSA samples (Figure S8). LUT and L3′S caused similar decreases in the ICD signal of BVD, with a slightly stronger impact of the metabolite (Figure 7E,F). For LUT, a weak ICD signal appeared in the spectrum without BVD, while it was not observed for L3′S (Figure S9). However, ICD bands around 275–280 nm were induced by the interaction of LUT and L3′S with the protein. MYR was the sole compound causing negligible changes in the ICD band of BVD (Figure 7G). In contrast, M3′S induced a marked decrease in a concentration-dependent manner (Figure 7H). Interestingly, in the presence of MYR, no ICD band was observed in the 275–280 nm range, unlike the other flavonoids tested.

**Figure 7:**
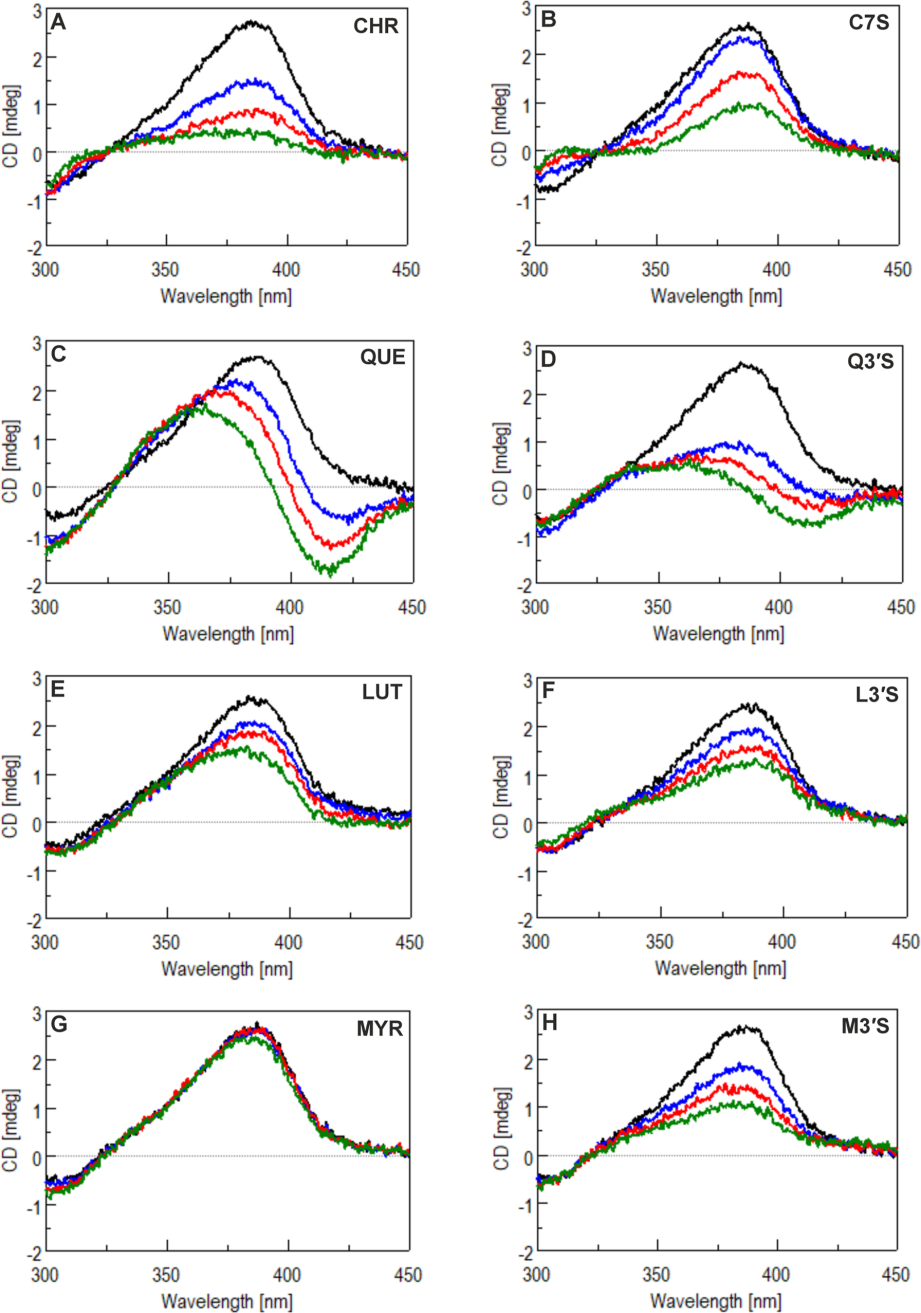
CD spectra of BVD + HSA (1 μM and 2 μM, respectively) in PBS (pH 7.4), in the absence and presence of increasing levels (0, 5, 10, or 20 μM) of flavonoids: CHR (A), C7S (B), QUE (C), Q3′S (D), LUT (E), L3′S (F), MYR (G), or M3′S (H).

### 3.4 Effects of Flavonoids on the Albumin Binding of BVD in the Presence of 10% FBS

The potential impacts of flavonoids on BVD–albumin interactions were also investigated in the presence of 10% FBS. FBS was diluted to 10-fold with PBS then the impacts of flavonoids (5, 20, and/or 50 μM) on the free fraction of BVD (5 μM) were examined. After ultracentrifugation, FBS (10%) induced an approximately 50% decrease in the BVD level of the supernate. Even at 50 μM concentration, MYR and M3′S had no statistically significant (*p* < 0.01) effects, they produced only slight decreases. Furthermore, CHR caused a moderate reduction while QUE, Q3′S, LUT, and L3′S induced marked decreases in BVD levels of the supernates (Figure 8). In contrast, C7S was the sole compound that resulted in gradual elevation in the free concentrations of the heme site marker. In the presence of 10% FBS, the impacts of L3′S vs LUT and M3′S vs MYR were similar. Furthermore, Q3′S had stronger effect than QUE, while CHR and C7S produced opposite outcomes regarding the modulation of BVD–BSA interaction (Figure 8). In the BVD–HSA model, each flavonoid increased free BVD levels (Figure 6A); however, we noticed the contrary (except C7S) with 10% FBS (Figure 8). Therefore, further measurements were also carried out with BSA in the presence of BVD and flavonoid sulfates: These experiments confirmed that C7S moderately increases, M3′S slightly reduces, while L3′S and mainly Q3′S strongly decrease the free fraction of BVD (Figure S11).

**Figure 8:**
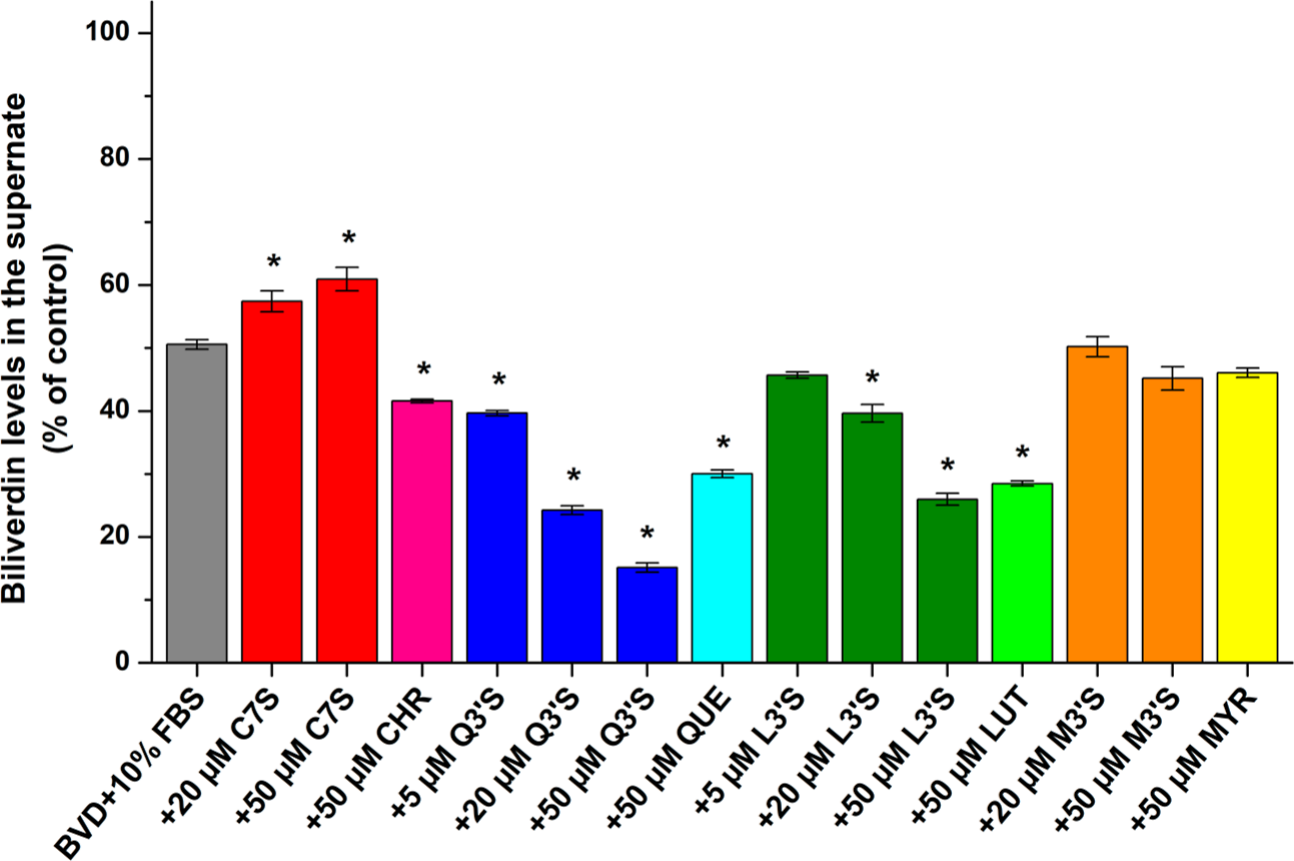
BVD (5 μM) levels in the supernates after ultracentrifugation (16 h, 170,000*g*, 20 °C) in the presence of 10% fetal bovine serum (FBS) without and with C7S, CHR, Q3′S, QUE, L3′S, LUT, M3′S, or MYR in PBS (pH 7.4). Data represent means ± SEM from three independent experiments (*p < 0.01), where the changes in BVD levels were compared to BVD (5 μM) ultracentrifuged without FBS and other ligands (100%).

The species-dependent variation in the albumin binding of BVD was also underlined by the observation that 2 μM HSA resulted in almost a 50% decrease in the free fraction of BVD (Figure 6A), whereas 30 μM BSA caused less than 45% decline (Figure S11). In addition, the evaluation of ultracentrifugation data (using eq 1) highlighted that the stability of BVD–HSA (log*K* = 5.78 ± 0.03) was more than 20-fold higher than that of the BVD–BSA complex (log*K* = 4.42 ± 0.02). In agreement with our data, previous spectroscopic studies have also suggested that the log*K* value of the BVD–HSA complex is around 5.5–6.1.^29,^ ^42^

## 4 Discussion

In ultracentrifugation experiments conducted with HSA, the following sequence was observed in relation to the displacing capability of flavonoids (20 μM) vs WAR: C7S > Q3′S > QUE ≥ L3′S > M3′S > LUT ≈ MYR > CHR (Figure 2A). These findings are consistent with the outcomes of earlier ultrafiltration experiments, in which sulfate metabolites exhibited stronger displacing effects than their respective aglycones, especially C7S and Q3′S induced remarkable increases in the free fraction of WAR.^14,16,20,21^ Even if the docking scores (Table S1) in our modeling studies do not provide a clear explanation, it can be speculated that the increased number of polar interactions, particularly salt bridges with Arg218 and Lys195 (Figure 4), observed for the sulfate conjugates may partially account for the overall higher WAR-displacing ability exhibited by the sulfate derivatives compared to the parent flavonoids. The superior displacing effect of C7S is in accordance with the previously reported, high binding constant of the C7S–HSA complex (log*K* = 5.9),^14^ while the log*K* values of the other aglycones and flavonoid sulfates examined have been reported in the 5.1–5.6 range.^14,16,20,21^ In addition, a recent study demonstrated that C7S can disrupt the albumin binding of ochratoxin A (a mycotoxin that binds to the Site I region of HSA with very high affinity).^30^

However, in spiked human serum samples, even 50 μM of flavonoid sulfates did not affect the free fraction of WAR. The concentration of HSA in human serum is approximately 600 μM: it has a large binding capacity,^1^ which may explain these results. Human studies showed nanomolar or low micromolar levels of flavonoid conjugates in the circulation, even after the peroral administration of large flavonoid doses (like dietary supplements).^10,12,43,44^ Thus, the current study provides circumstantial evidence that, even if their very high levels appear in the circulation, it is unlikely that flavonoids or their metabolites could considerably displace Site I ligand drugs from HSA. This observation is in agreement with earlier reports, suggesting that the pharmacokinetic interactions of certain drugs (e.g., phenylbutazone) with WAR are not resulted from the displacement of WAR from albumin but are caused by other interactions (e.g., inhibition of CYP2C9 enzyme).^2^

In the presence of 20 μM flavonoid concentrations, the flavonoid-induced decrease in the fluorescence emission signal of WAR (Figure 3) suggests the following order of displacing ability: C7S > Q3′S > QUE ≥ LUT > L3′S > M3′S > MYR > CHR. This is mostly in agreement with the results of ultracentrifugation experiments (Figure 2A); nevertheless, we emphasize two important differences. LUT caused a stronger decrease in the emission signal of WAR than L3′S and M3′S (Figure 3), whereas ultracentrifugation experiments demonstrated its considerably weaker displacing effects compared to sulfate metabolites (Figure 2A). Furthermore, even low levels of flavonoids (5 μM) caused a marked reduction in the fluorescence signal of WAR; however, in ultracentrifugation experiments, some of these compounds did not show relevant effects even at 20 μM concentrations. Based on these data, it is reasonable to hypothesize that certain flavonoids can decrease the emission signal of WAR even without considerable displacement from the protein, which is likely the result of the reduced emission signal of the WAR–HSA complex. In our earlier report, we demonstrated that the cooperative binding of heme site markers to HSA can strongly decrease the emission signal of WAR, regardless the changes in the bound fraction of the Site I marker drug;^29^ and some studies suggest that flavonoids and WAR may be able to simultaneously occupy Site I.^45,46^ As another possible explanation, flavonoids may have further binding site(s) on the protein, where cooperative binding with WAR can influence the emission signal of the WAR–HSA complex. For example, a recent study (applying competition experiments with specific fluorescent dyes) suggest that Q3′S and chrysin have secondary binding sites in Site II.^19^ Our results suggest that the fluorescence spectroscopic method used in the current study gives an acceptable approximation to compare the displacing ability of structurally similar flavonoids; however, it does not ensure strong evidence and provides an imprecise estimation of displacing ability.

Another interesting practical issue is the potential interactions of flavonoids in cell culture media, which typically contain 10% FBS. In this model, L3′S (50 μM) doubled the free fraction of WAR; nevertheless, at 20 μM concentration, flavonoids caused no or only moderate changes. These results emphasize that high flavonoid levels can affect the albumin binding of Site I ligands in cell culture media, possibly resulting in pharmacokinetic interactions in cell experiments; however, lower levels of flavonoids are likely not relevant from this perspective. Nevertheless, we need to emphasize the species-dependent differences in the displacing ability of flavonoids depending on HSA or BSA applied in the experiments (Figures 2A, Figures 5, and S5).

The primary binding sites of flavonoids (Site I) and BVD (heme site) are allosterically coupled.^1^ Therefore, as a result of the simultaneous/cooperative binding of a Site I ligand and BVD to albumin, the binding affinity of BVD can increase, decrease, or even remain unchanged. In accordance with these possibilities, the Site I marker drugs tested showed examples of all three outcomes (Figure 6B). Interestingly, in our earlier study, we demonstrated that BVD can stabilize the WAR–HSA complex.^29^ This impact seems to work vice versa, because the decreased level of BVD in the supernatant means its increased HSA-bound fraction. In ultracentrifugation studies with HSA, each flavonoid increased the free fraction of BVD, with the following order observed in the presence of 20 μM flavonoid concentrations: Q3′S > L3′S > C7S > CHR > M3′S ≈ LUT > MYR > QUE. Despite the marked negative modulatory effects of Q3′S, L3′S, and C7S on BVD–HSA interaction (Figure 6A), they did not modify the free fraction of BVD in spiked human serum (Figure 6C). Therefore, it seems to be unlikely that even high levels of flavonoids and/or their metabolites could affect the albumin binding of heme site ligands in the circulation.

In the circular dichroism spectra of proteins, there are no measurable signals above 300 nm. Between 250 and 300 nm, a band usually appears due to the aromatic side chains (Figure S6), and below 250 nm, a band system that is characteristic of the protein’s secondary structure appears.^47^ When a chromophore-containing, achiral molecule binds to a protein, the electron transitions become chirally perturbed upon excitation, resulting in the phenomenon known as ICD, which appears within a given spectral range.^5^ The appearance of an isoelliptic point on the spectra during titration (Figure S6) indicates the 1:1 stoichiometry of the BVD–HSA complex, suggesting that the ligand binds selectively to subdomain IB. Q3′S, L3′S, and M3′S caused larger changes in the ICD signal of BVD compared to their parent aglycones, while CHR produced stronger impact than C7S (Figure 7). Based on these results, CD spectroscopy seems to be suitable for the evaluation of the displacing effects of flavonoids; nevertheless, the flavonoid-induced background signals and band splitting (e.g., QUE, Q3′S, and LUT; Figures S7–S10) make the precise quantitative evaluation problematic. Furthermore, we do not have any explanation for why CHR caused larger decreases in the ICD band of BVD compared to C7S (Figure 7A,B), while the metabolite demonstrated its slightly stronger displacing effect in ultracentrifugation experiments (Figure 6A).

Based on ultracentrifugation studies, most of the flavonoids (mainly Q3′S and L3′S) can stabilize the BVD–BSA complex; however, C7S caused an opposite effect (Figures 8 and S11). These data emphasize again the species-dependent differences in flavonoid–albumin interactions, where not only the extent of the impact but even its direction can be distinct depending on the presence of HSA or BSA. Some flavonoids (e.g., Q3′S, QUE, L3′S, and LUT) can strongly modulate the free fraction of BVD in the presence of 10% FBS, which can likely affect the results of cell experiments where both flavonoids and certain heme site ligands are applied. Herein, we examined BVD as a well-established heme site marker, but flavonoids may cause different effects during their cooperative binding to albumin with other heme site ligands.

The ultracentrifugation technique has some limitations because sedimentation, back diffusion, or entrapment of certain compounds in the chylomicron layer (e.g., when plasma samples are centrifuged) can cause quantitative discrepancies.^22,48^ Nevertheless, in our experiments with HSA, human serum, BSA, and FBS, the results were in good agreement with each other and with the previously reported data, demonstrating the suitability of the ultracentrifugation technique to evaluate these ligand–protein interactions.

## 5 Conclusions

In summary, the effects of C7S, Q3′S, L3′S, M3′S, and their aglycones on the albumin binding of WAR (Site I) and BVD (heme site) were examined. WAR binds to HSA with a slightly higher affinity than to BSA; however, the stability of BVD–HSA complex is more than 20-fold higher compared to BVD–BSA. Flavonoid sulfates can considerably displace WAR from both HSA and BSA: C7S and Q3′S showed the strongest effects in the presence of HSA, while L3′S and Q3′S proved to be the most effective competitors when BSA was applied. BVD–HSA interaction can be markedly disrupted by Q3′S, L3′S, and C7S; in contrast, Q3′S, QUE, L3′S, and LUT strongly increased the stability of the BVD–BSA complex. These observations emphasize the significant species-dependent variations in the displacing and modulatory effects of flavonoids regarding ligand–albumin interactions. Flavonoid sulfates did not affect the free fraction of WAR and BVD in spiked human serum samples, strongly suggesting that flavonoids cannot modify the albumin binding of Site I and heme site ligands in the human circulation. However, in the experiments with 10% FBS, flavonoids elevated the free fraction of WAR while decreased the free fraction of BVD. Therefore, it is reasonable to assume that certain flavonoids can influence the results of cell experiments, due to the marked changes induced in the albumin-bound fraction of other ligands. The present study provides a better insight and gives a good starting point to evaluate the possible flavonoid-induced changes in drug–albumin interactions, considering both theoretical and practical perspectives.

## Supplementary Material



## Notes

Fluorescence emission spectra of WAR in the presence of increasing concentrations of HSA (Figure S1). Representative fluorescence emission spectra of WAR + HSA in the presence of increasing concentrations of flavonoids (Figure S2). Docking pose of WAR compared to its crystallographic pose (Figure S3). Docking scores of the set of tested flavonoids (Table S1). Binding poses of WAR and flavonoids within the binding pocket of HSA (Site I) after docking simulations (Figure S4). WAR levels in the supernate after ultracentrifugation in the presence of BSA without and with flavonoids (Figure S5). CD and UV spectra of HSA in the absence and presence of increasing concentrations of BVD (Figure S6). CD and UV spectra of BVD + HSA in the absence and presence flavonoids; and spectra of HSA without and with flavonoids (Figures S7–S11). BVD levels in the supernate after ultracentrifugation in the presence of BSA without and with flavonoids (Figure S11) (PDF)
