# Supplementary material for: Effects of Sulfate Metabolites of Chrysin, Quercetin, Luteolin, and Myricetin on the Albumin Binding of Warfarin (Site I) and Biliverdin (Heme Site): from Theoretical to Practical Considerations
Source: ACS Omega. 2026 Mar 25;11(13):21344–56. doi: 10.1021/acsomega.6c01121 (PMC13063089; doi:10.1021/acsomega.6c01121)
Supplement: Supplementary file 1 [file ao6c01121_si_001.pdf]

Effects of sulfate metabolites of chrysin, quercetin, luteolin, and myricetin on the albumin binding of warfarin (Site I) and biliverdin (heme site): From theoretical to practical considerations

SUPPLEMENTARY MATERIALS

Miklós Poór [1,2,*], Lorenzo Pedroni [3], Dávid Csabai [1], Dávid Hesszenberger [1], Anikó Lajtai [1], Patrik Gömbös [4], Tamás Huber [5], Szilvia Barkó [5], András Lukács [5], Paul A. Kroon [6], Kateřina Valentová [7], Luca Dellafiora [3], Péter Horváth [8]

[1] Department of Laboratory Medicine, Medical School, University of Pécs, Ifjúság útja 13, H-7624 Pécs, Hungary

[2] Molecular Medicine Research Group, János Szentágothai Research Centre, University of Pécs, Ifjúság útja 20, H-7624 Pécs, Hungary

[3] Department of Food and Drug, University of Parma, Via G.P. Usberti 27/A, 43124 Parma, Italy

[4] Agribiotechnology and Precision Breeding for Food Security National Laboratory, Institute of Physiology and Nutrition, Department of Physiology and Animal Health, Hungarian University of Agriculture and Life Sciences, Guba Sándor u. 40, H-7400 Kaposvár, Hungary

[5] Department of Biophysics, Medical School, University of Pécs, Szigeti út 12, H-7624 Pécs, Hungary

[6] Food, Microbiome & Health Programme, Quadram Institute Bioscience, Norwich Research Park, Norwich, Norfolk NR4 7UQ, UK

[7] Institute of Microbiology of the Czech Academy of Sciences, Vídeňská 1083, CZ-142 00 Prague, Czech Republic

[8] Department of Pharmaceutical Chemistry, Semmelweis University, Hőgyes Endre u. 9, H-1092 Budapest, Hungary

*Corresponding author:

Dr. Miklós Poór, Department of Laboratory Medicine, Medical School, University of Pécs, Ifjúság útja 13, H-7624 Pécs, Hungary; e-mail: poor.miklos@pte.hu

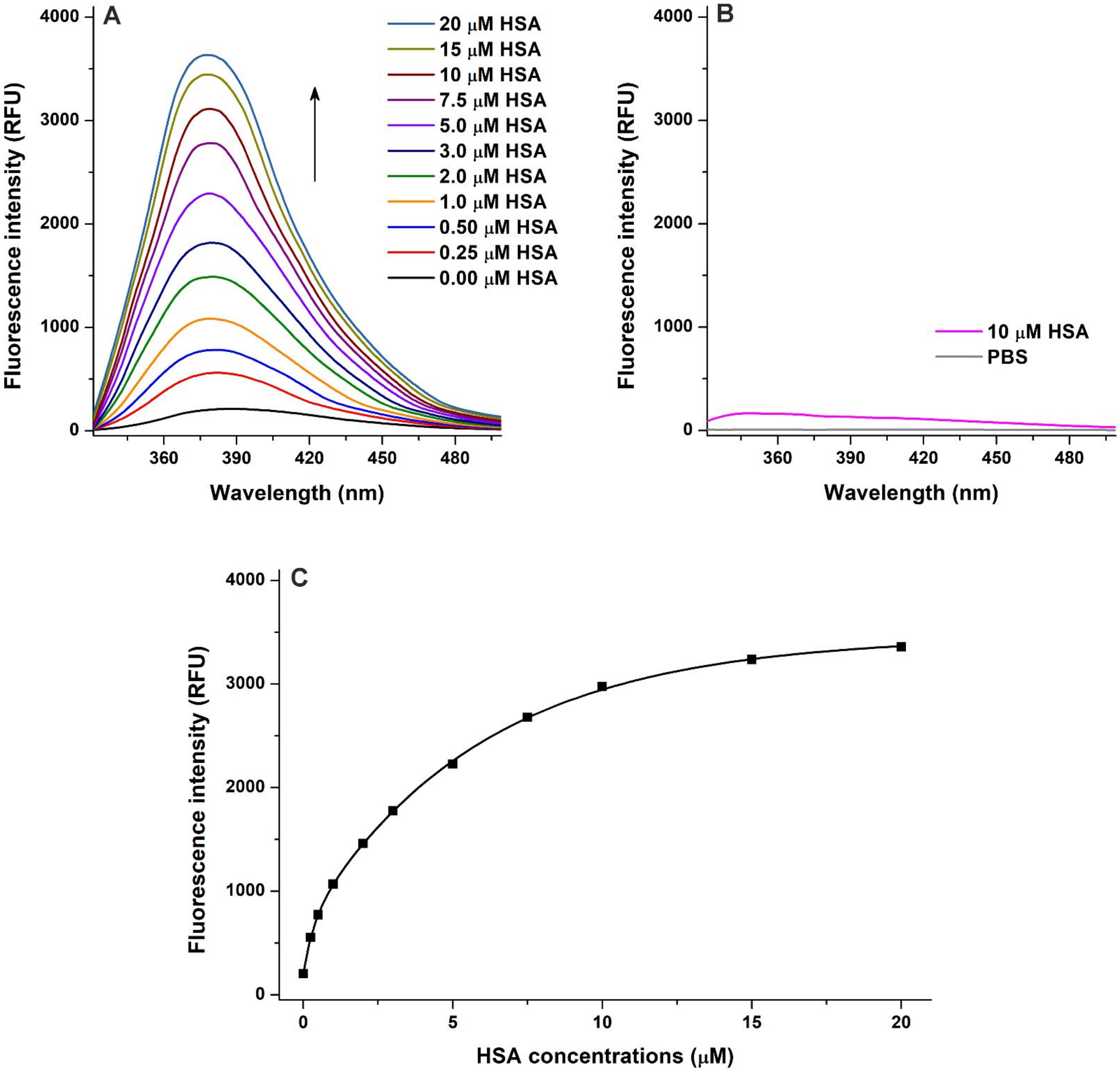

Figure S1: Fluorescence emission spectra of WAR (1 μM) in the presence of increasing HSA concentrations (0–20 μM) in PBS (pH 7.4; λ_{ex} = 317 nm; ex slit: 5 nm, em slit: 10 nm) (**A**). Background signals of PBS buffer and HSA (10 μM) under the same experimental conditions (**B**). HSA-induced elevation of the fluorescence emission signal of WAR (λ_{em} = 380 nm), after the correction of the background signals of HSA (**C**).

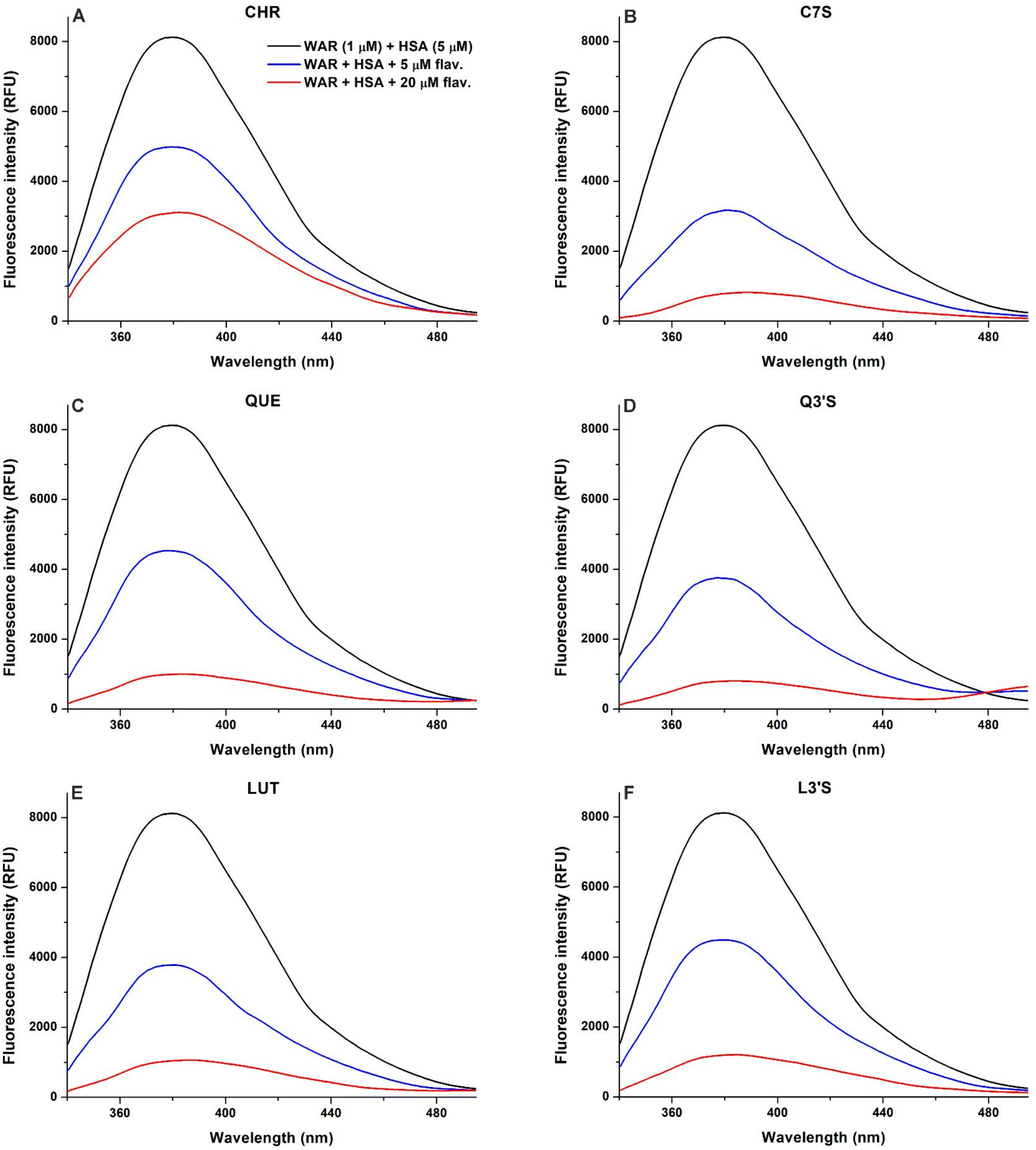

Continued on the next page.

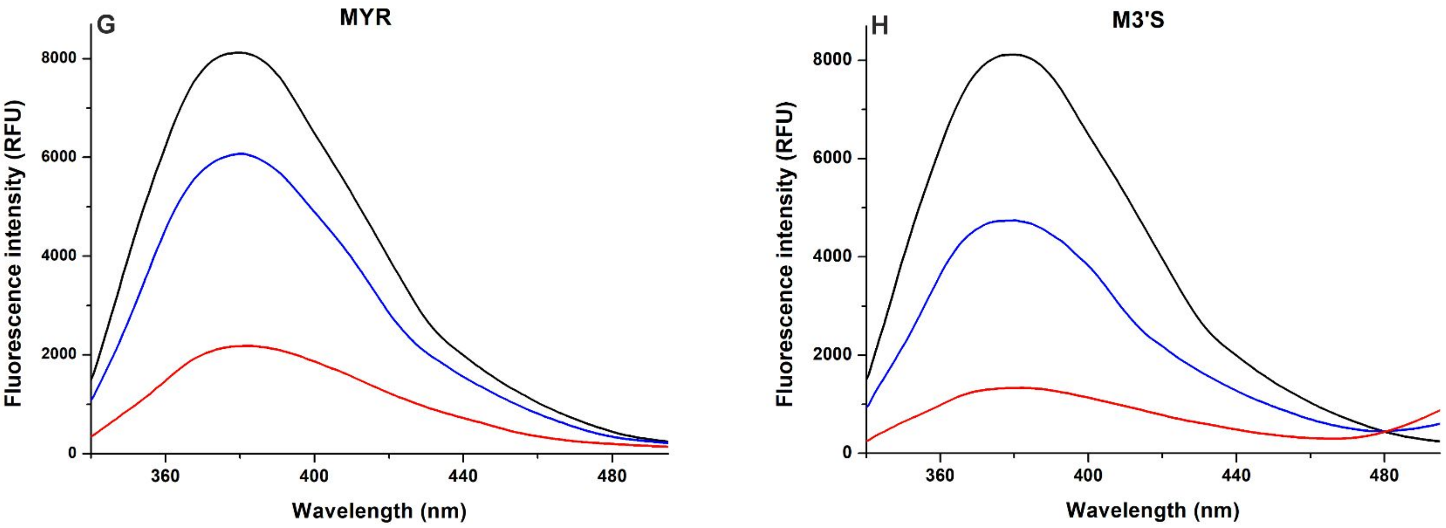

Figure S2: Representative fluorescence emission spectra of warfarin + HSA (1 μM and 5 μM, respectively) in PBS (pH 7.4; λ_{ex} = 317 nm; ex slit: 10 nm, em slit: 10 nm), in the presence of increasing concentrations of flavonoids (0, 5, or 20 μM): CHR (**A**), C7S (**B**), QUE (**C**), Q3′S (**D**), LUT (**E**), L3′S (**F**), MYR (**G**), or M3′S (**H**).

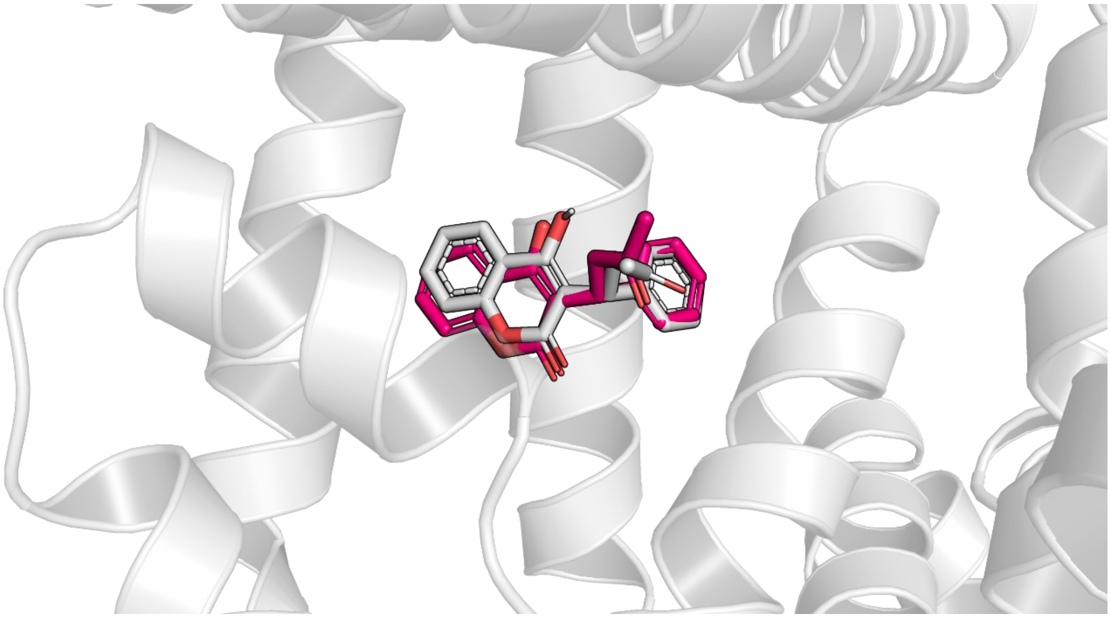

Figure S3: HSA is reported as white transparent cartoon. WAR docking pose is shown as white sticks while the crystallographic pose as magenta sticks. It can be appreciated the nearly perfect superimposition between the two poses.

Table S1: Docking scores of the set of tested flavonoids.

Ligand	Docking Score (units)
**QUE**	**61.81**
**Q3'S**	**60.01**
**CHR**	**44.19**
**C7S**	**53.91**
**MYR**	**59.80**
**M3'S**	**60.52**
**LUT**	**58.74**
**L3'S**	**58.74**
**WAR**	**70.01**

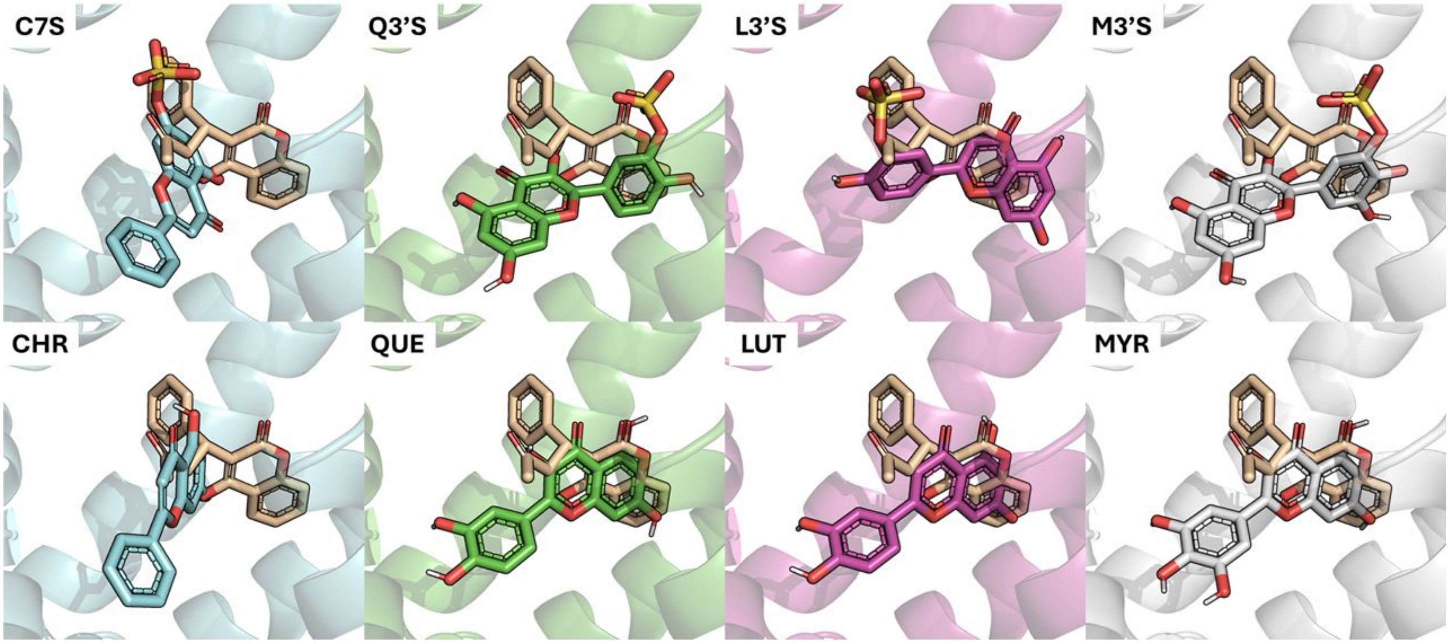

Figure S4: Binding poses of WAR and flavonoids within the binding pocket of HSA (Site I) after docking simulations. Top panels show the flavonoids sulfated derivatives (C7S, Q3′S, L3′S, and M3′S), while the corresponding non-sulfated flavonoids (CHR, QUE, LUT, and MYR) are demonstrated in the bottom panels. The protein is displayed as a transparent cartoon, and the ligands are shown as colored sticks with WAR always represented as light brown sticks. The binding site of each flavonoid – at least partially – overlap with the binding pose of WAR.

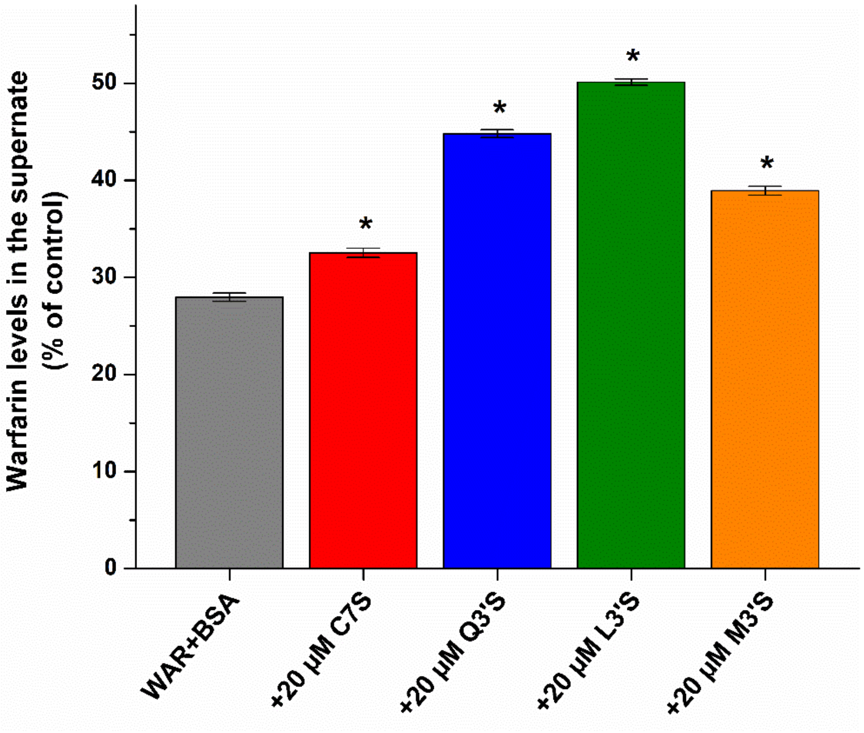

Figure S5: Warfarin (WAR, 1 µM) levels in the supernate after ultracentrifugation (16 h, 170,000 g, 20 °C) in the presence of bovine serum albumin (BSA, 10 µM) without and with chrysin-7-*O*-sulfate (C7S), quercetin-3′-*O*-sulfate (Q3′S), luteolin-3′-*O*-sulfate (L3′S) or myricetin-3′-*O*-sulfate (M3′S) in PBS (pH 7.4). Data represent means ± SEM from three independent experiments (*$p < 0.01$), where the changes in WAR levels were compared to WAR (1 µM) ultracentrifuged without the protein and other ligands (100%).

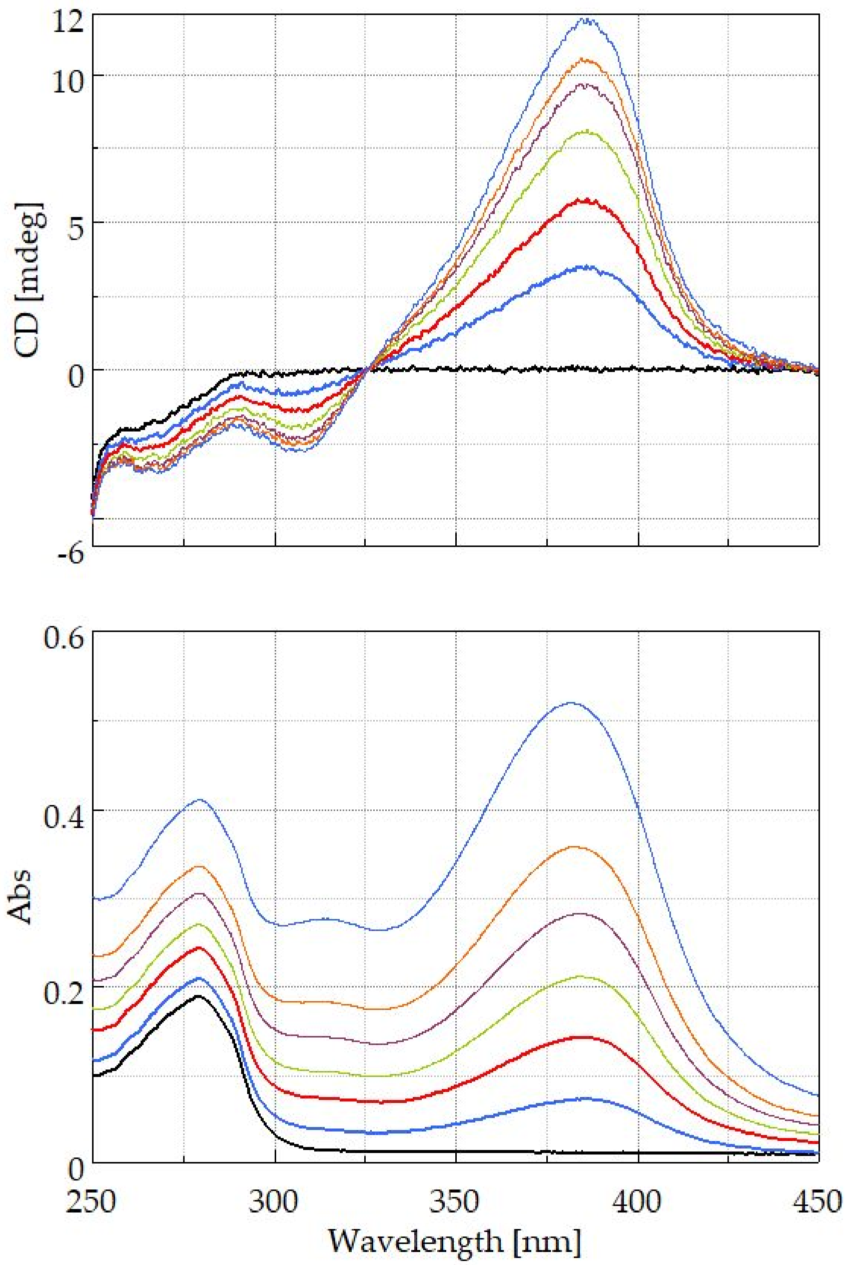

5

HSA (3 µM); HSA + 0.67 µM BVD; HSA + 1.33 µM BVD; HSA + 1.66 µM BVD; HSA + 2.66 µM BVD; HSA + 3.32 µM BVD; HSA + 5.31 µM BVD

Figure S6: CD and UV spectra of HSA (3 µM) in the absence and presence of increasing concentrations of BVD in PBS (pH 7.4).

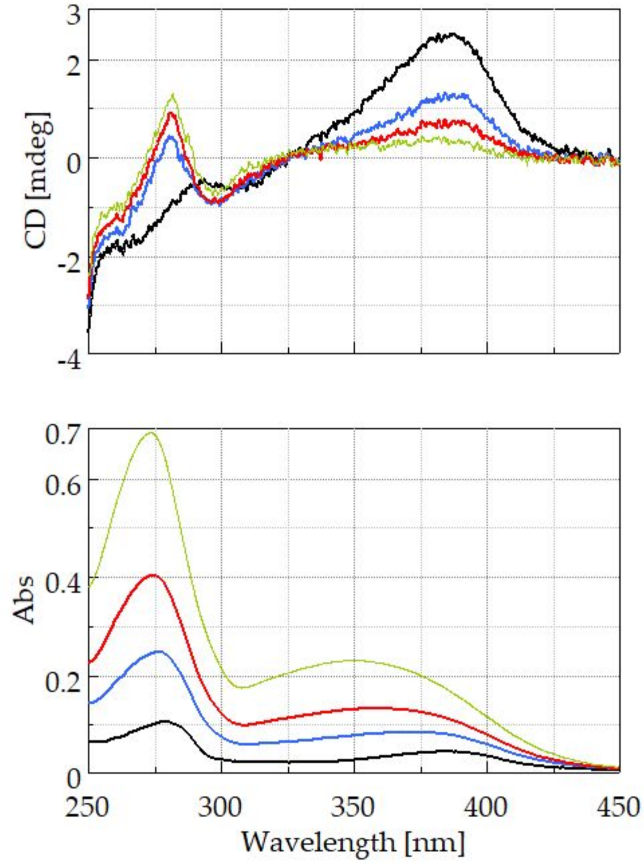

BVD (1 µM) + HSA (2 µM); BVD + HSA + 5 µM CHR; BVD + HSA + 10 µM CHR; BVD + HSA + 20 µM CHR

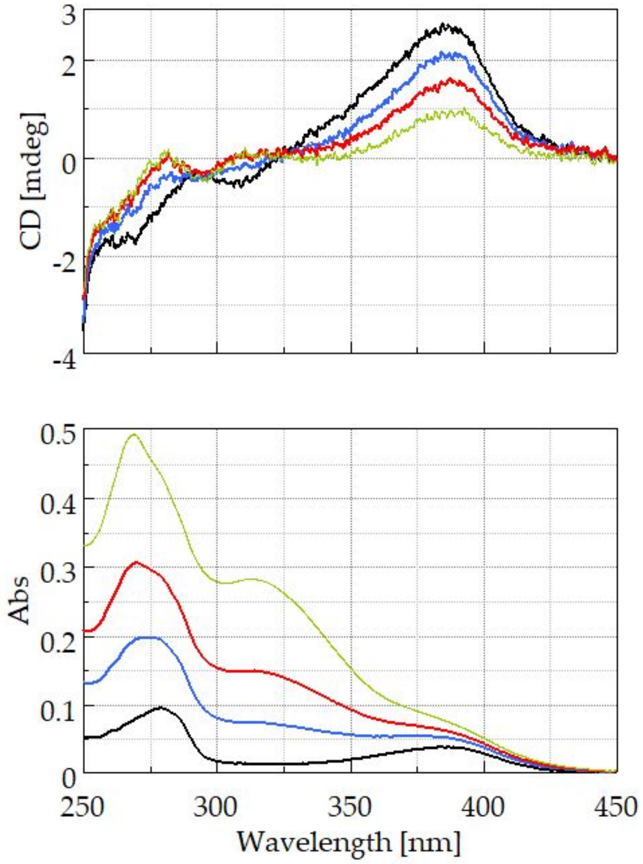

BVD (1 µM) + HSA (2 µM); BVD + HSA + 5 µM C7S; BVD + HSA + 10 µM C7S; BVD + HSA + 20 µM C7S

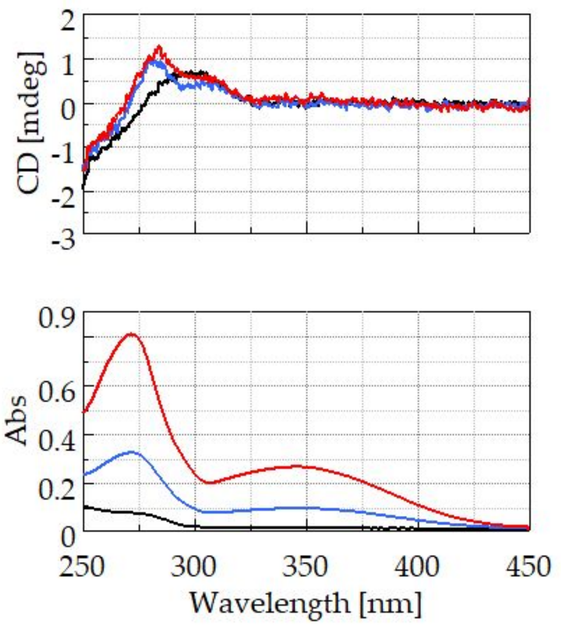

HSA (2 µM); HSA + 5 µM CHR; HSA + 20 µM CHR

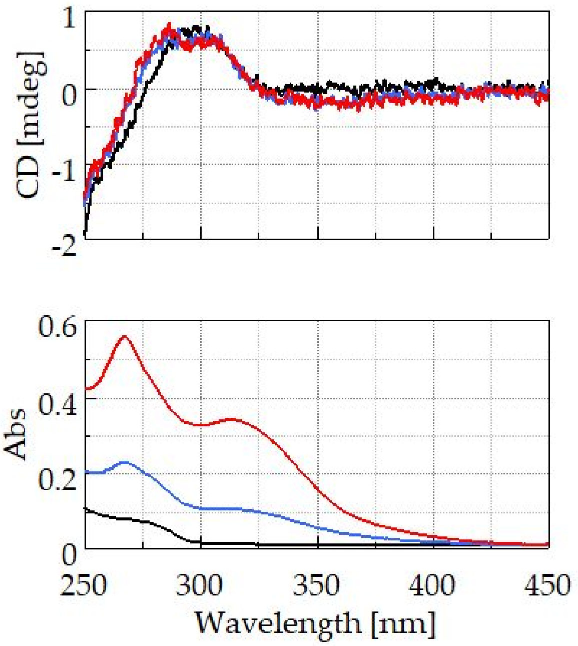

HSA (2 µM); HSA + 5 µM C7S; HSA + 20 µM C7S

Figure S7: Top panels: CD and UV spectra of BVD + HSA (1 µM and 2 µM, respectively) in the absence and presence of increasing levels of CHR or C7S in PBS (pH 7.4). Bottom panels: CD and UV spectra of HSA (2 µM) without and with CHR or C7S.

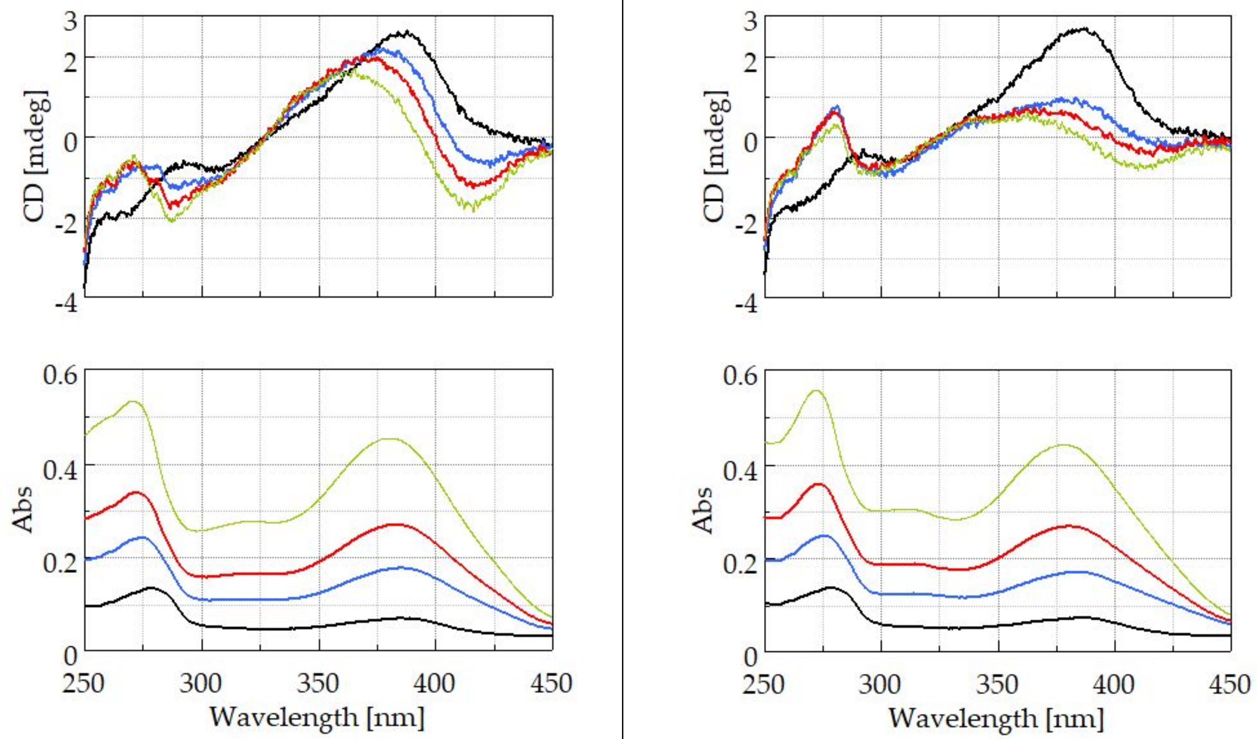

BVD (1 μM) + HSA (2 μM); BVD + HSA + 5 μM QUE; BVD + HSA + 10 μM QUE; BVD + HSA + 20 μM QUE

BVD (1 μM) + HSA (2 μM); BVD + HSA + 5 μM Q3′S; BVD + HSA + 10 μM Q3′S; BVD + HSA + 20 μM Q3′S

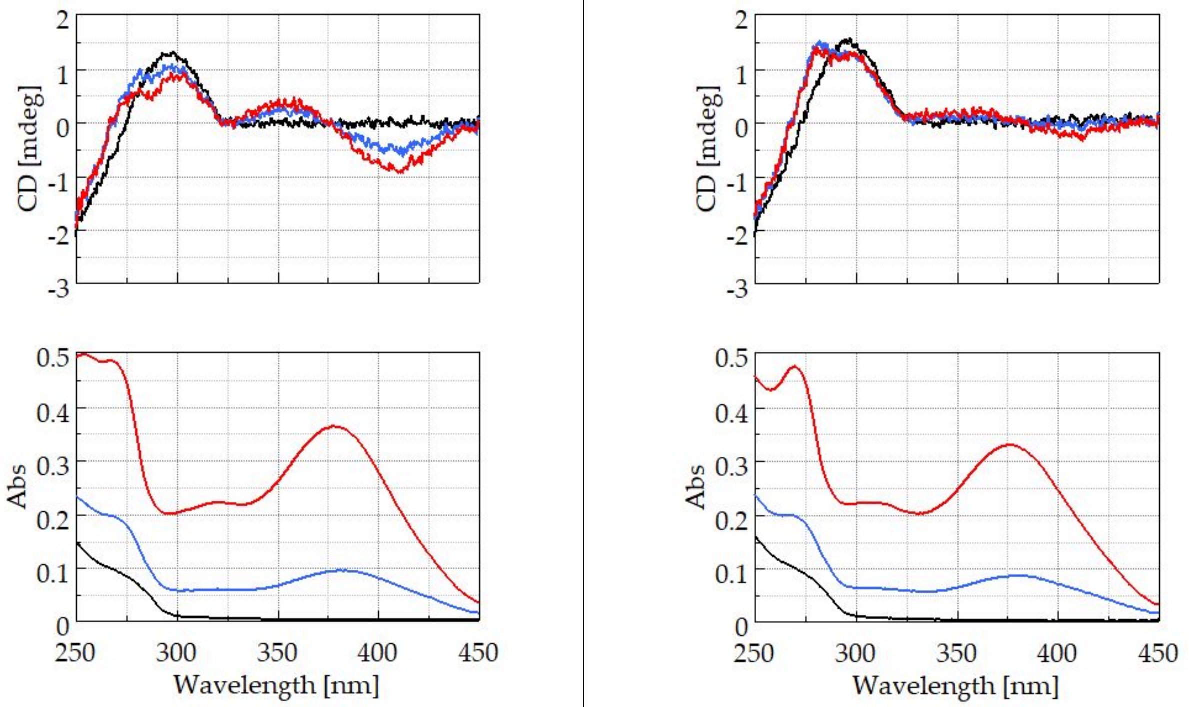

HSA (2 μM); HSA + 5 μM QUE; HSA + 20 μM QUE

HSA (2 μM); HSA + 5 μM Q3′S; HSA + 20 μM Q3′S

Figure S8: Top panels: CD and UV spectra of BVD + HSA (1 μM and 2 μM, respectively) in the absence and presence of increasing levels of QUE or Q3′S in PBS (pH 7.4). Bottom panels: CD and UV spectra of HSA (2 μM) without and with QUE or Q3′S.

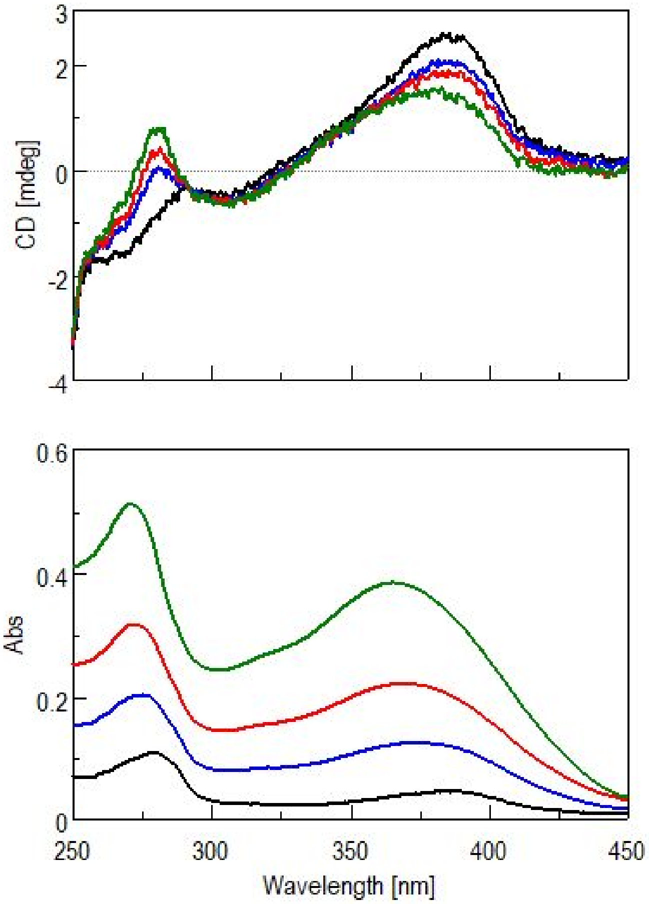

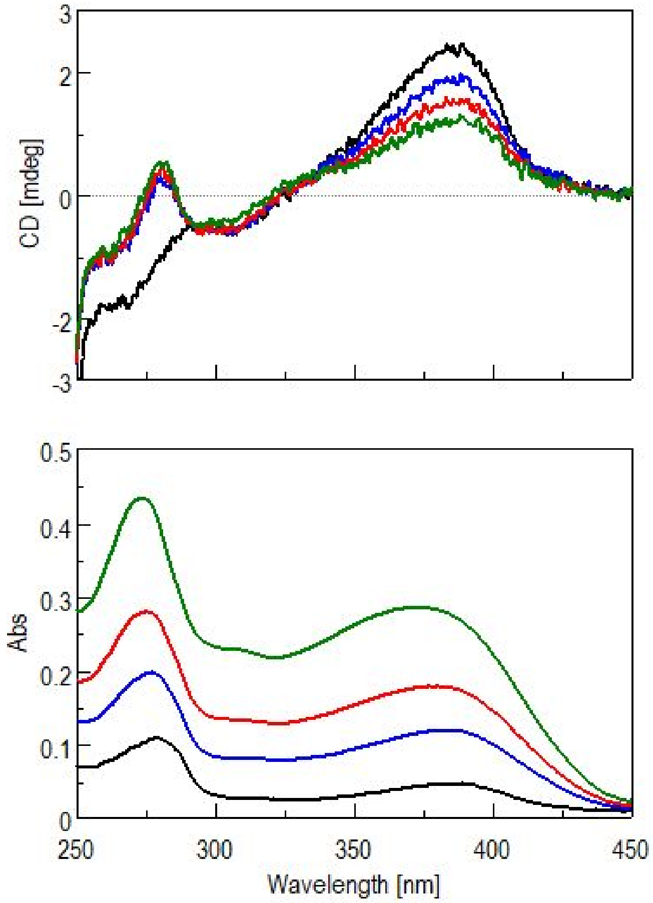

BVD (1 μM) + HSA (2 μM); BVD + HSA + 5 μM LUT; BVD + HSA + 10 μM LUT; BVD + HSA + 20 μM LUT

BVD (1 μM) + HSA (2 μM); BVD + HSA + 5 μM L3′S; BVD + HSA + 10 μM L3′S; BVD + HSA + 20 μM L3′S

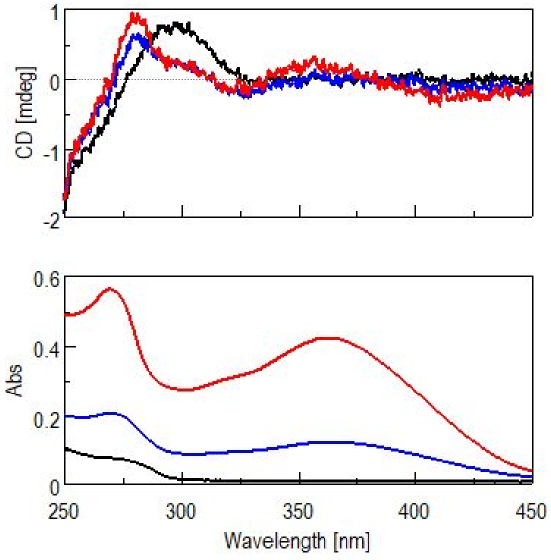

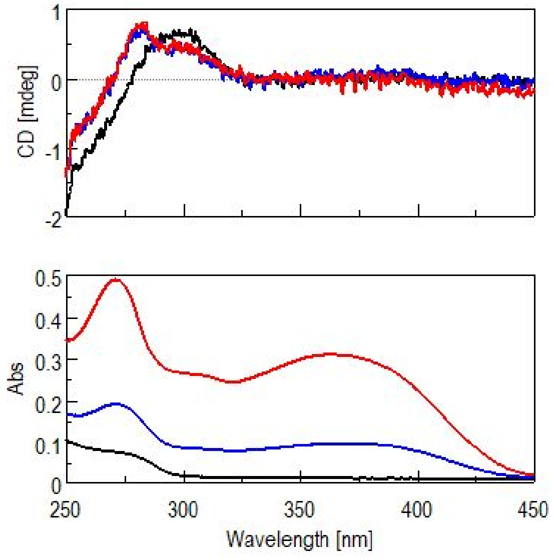

HSA (2 μM); HSA + 5 μM LUT; HSA + 20 μM LUT

HSA (2 μM); HSA + 5 μM L3′S; HSA + 20 μM L3′S

Figure S9: Top panels: CD and UV spectra of BVD + HSA (1 μM and 2 μM, respectively) in the absence and presence of increasing levels of LUT or L3′S in PBS (pH 7.4). Bottom panels: CD and UV spectra of HSA (2 μM) without and with LUT or L3′S.

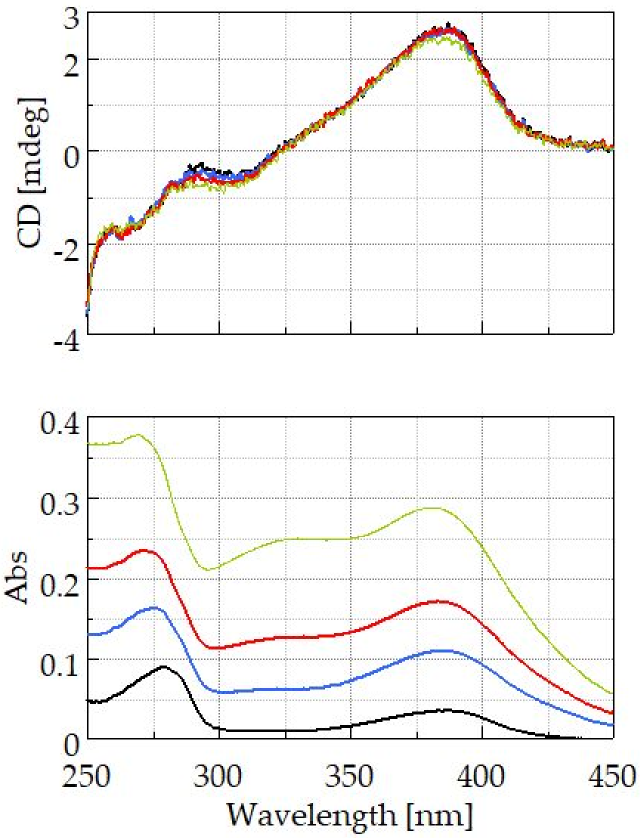

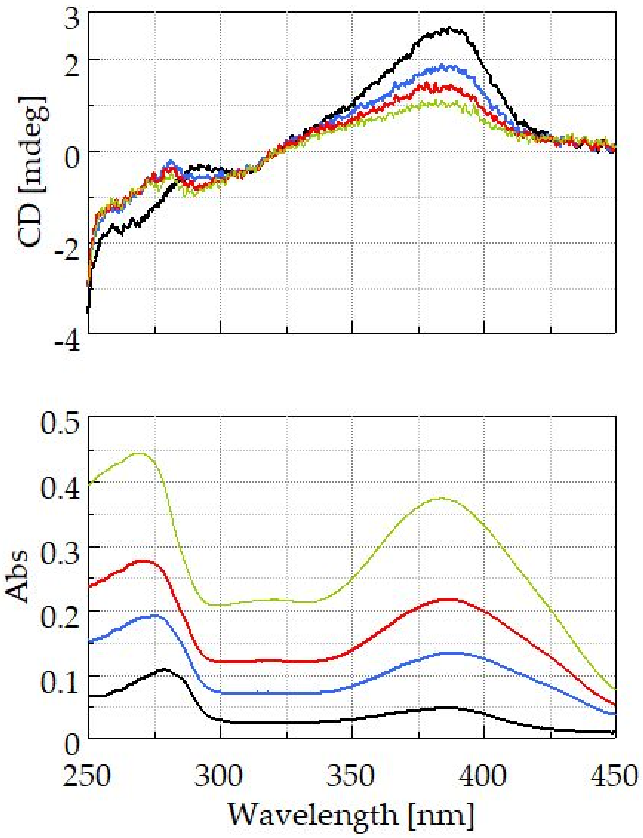

BVD (1 µM) + HSA (2 µM); BVD + HSA + 5 µM MYR; BVD + HSA + 10 µM MYR; BVD + HSA + 20 µM MYR

BVD (1 µM) + HSA (2 µM); BVD + HSA + 5 µM M3′S; BVD + HSA + 10 µM M3′S; BVD + HSA + 20 µM M3′S

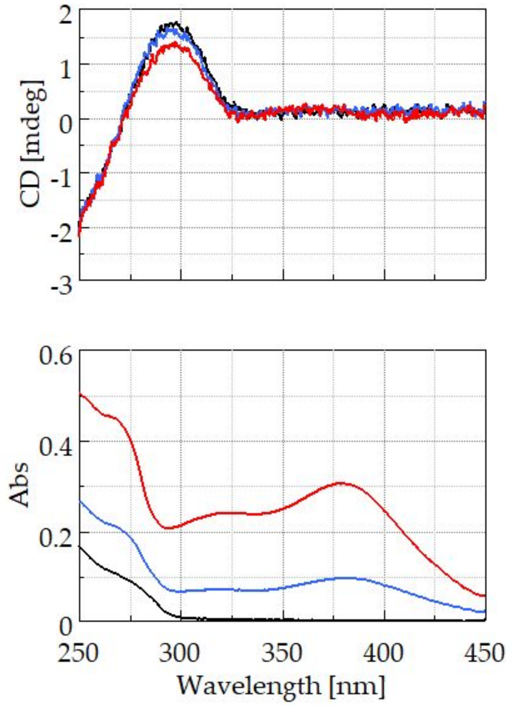

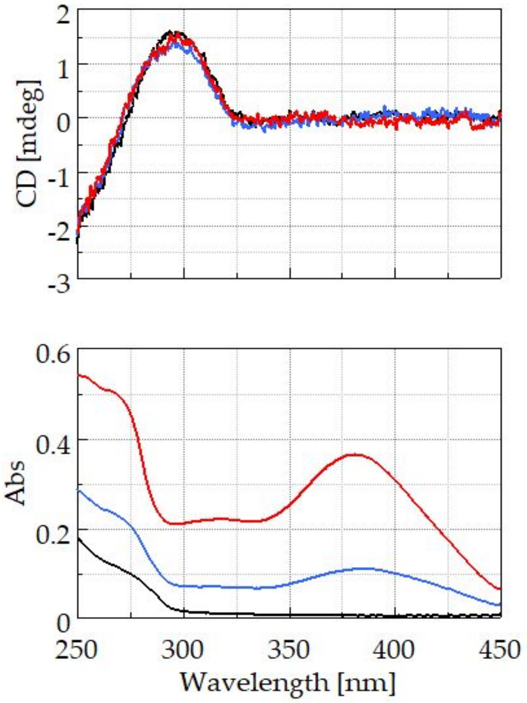

HSA (2 µM); HSA + 5 µM MYR; HSA + 20 µM MYR

HSA (2 µM); HSA + 5 µM M3′S; HSA + 20 µM M3′S

Figure S10: Top panels: CD and UV spectra of BVD + HSA (1 µM and 2 µM, respectively) in the absence and presence of increasing levels of MYR or M3′S in PBS (pH 7.4). Bottom panels: CD and UV spectra of HSA (2 µM) without and with MYR or M3′S.

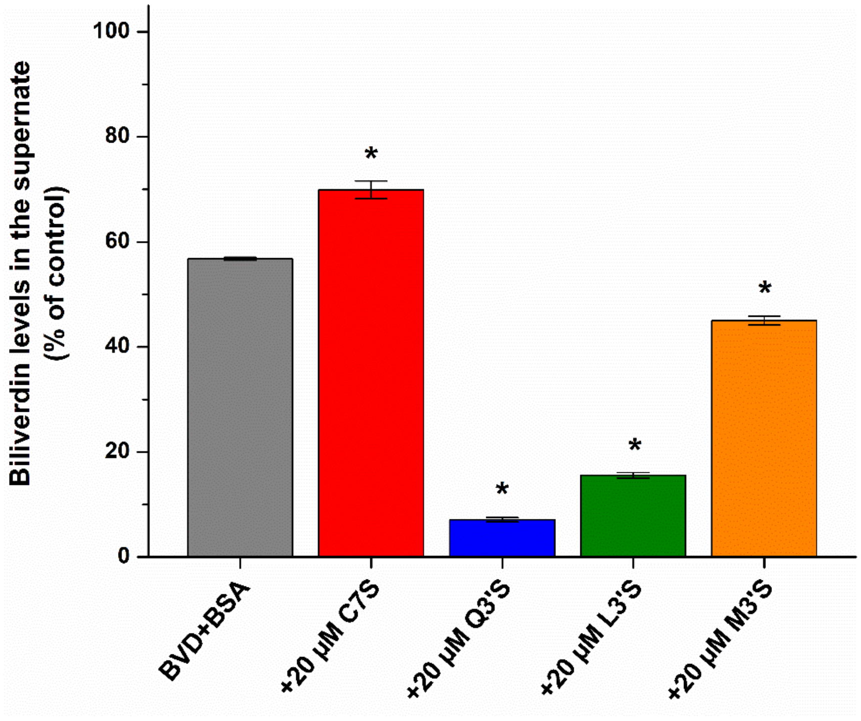

Figure S11: Biliverdin (BVD, 1 µM) levels in the supernate after ultracentrifugation (16 h, 170,000 g, 20 °C) in the presence of bovine serum albumin (BSA, 30 µM) without and with chrysin-7-*O*-sulfate (C7S), quercetin-3′-*O*-sulfate (Q3′S), luteolin-3′-*O*-sulfate (L3′S) or myricetin-3′-*O*-sulfate (M3′S) in PBS (pH 7.4). Data represent means ± SEM from three independent experiments (*p < 0.01), where the changes in BVD levels were compared to BVD (1 µM) ultracentrifuged without the protein and other ligands (100%).
